# Global burden of atrial fibrillation/flutter attributable to a high body mass index (HBMI) from 1990–2021

**DOI:** 10.1186/s12872-025-05125-5

**Published:** 2025-10-14

**Authors:** Dongyang Xu, Xiaoxue Guo, Zhaoyang Wei, Guanghui Liu, Xishu Wang, Ying Sun, Tong Wang, Zhiguo Zhang

**Affiliations:** Department of Cardiology, The First Hospital of Jilin University, Changchun, Jilin Province 130021 China

**Keywords:** High body mass index, Atrial fibrillation, Obesity, Epidemiology, Global burden of disease, Public health

## Abstract

**Aims:**

This study leveraged the Global Burden of Disease (GBD) database to assess sex- and region-specific trends in obesity-attributable atrial fibrillation and atrial flutter (AF/AFL) over the past three decades. We aimed to unravel temporal dynamics to inform evidence-based prevention and clinical management strategies.

**Methods:**

Using 2021 GBD data, we evaluated the AF/AFL burden attributable to high body mass index (HBMI). Our analysis included deaths, age-standardized rates (ASR), disability-adjusted life years (DALYs), and estimated annual percentage changes (EAPC), stratified by age group, gender, Sociodemographic Index (SDI), and region. We also assessed health inequalities using the Gini coefficient and conducted hypothesis testing to examine relationships between mortality, disability rates, and gender from 1990 to 2021.

**Results:**

In 2021, HBMI-related AF/AFL caused 27,236 deaths and 724,573 DALYs globally. These figures represent increases of 66.7% and 272.5%, respectively, compared to 1990. The age-standardized mortality rate (ASMR) and DALY rate rose by 66.7% and 67.8%. Nearly half of the disease burden occurred in high and upper-middle SDI regions; however, other SDI regions are experiencing a rapid increase in burden. Notably, elderly women aged 65 + bore the highest disease burden. The Gini index for DALY rates declined from 0.45 in 1990 to 0.35 in 2020, indicating reduced health inequality. While women had a higher overall burden, men exhibited faster growth in mortality and disability rates. Over the past 30 years, the EAPC for DALYs and mortality in men was 1.76 and 1.85 times higher, respectively, than in women. Critically, in high-SDI regions, men’s absolute disability burden and rates have surpassed women’s across all age groups.

**Conclusion:**

Disparities in the HBMI-attributable AF/AFL burden (HAB) across sociodemographic strata have narrowed significantly over the past three decades, reflecting progressive mitigation of structural health inequities. Economic advancement propagates the HAB across the development spectrum, and temporal analysis confirms this trajectory. The current burden profile observed in high-SDI regions suggests the future epidemiological path for lower-SDI settings. GBD projections indicate an impending reversal of the AF/AFL burden sex differential. Accelerating male-predominant obesity trends are poised to drive disproportionate burden escalation in coming decades. Targeted screening and early therapeutic intervention in young obese cohorts may mitigate future burden escalation among aging populations.

**Supplementary Information:**

The online version contains supplementary material available at 10.1186/s12872-025-05125-5.

## Introduction

Atrial fibrillation and flutter (AF/AFL), the most prevalent clinical arrhythmias, exhibit a consistently increasing global disease burden. Epidemiological projections indicate that the number of AF cases in the United States is expected to reach 6–12 million by 2050, while in Europe, the AF patient population may expand to 17–19 million cases by 2026 [1]. These cardiac arrhythmias not only substantially elevate the risk of acute events such as stroke and sudden cardiac death but also demonstrate strong pathophysiological associations with the development and progression of chronic conditions, including chronic kidney disease and heart failure [2]. Consequently, this dual burden has emerged as a critical public health challenge [3].

Over the past five decades, the global prevalence of obesity has increased dramatically, reaching epidemic proportions [4]. The World Health Organization (WHO) designates body mass index (BMI) as the primary screening indicator for overweight and obesity. Compelling evidence conclusively demonstrates that high body mass index (HBMI) constitutes an independent risk factor for AF/AFL development [2]. The underlying pathological mechanisms involve multiple pathways: HBMI contributes to AF/AFL pathogenesis by inducing metabolic dysregulation, promoting systemic complications, and enhancing chronic inflammatory responses and oxidative stress. These processes ultimately lead to cardiac structural and electrical remodeling that facilitates AF initiation and perpetuation. Furthermore, recent prospective cohort studies and Mendelian randomization analyses provide high-level evidence supporting the HBMI-AF association [5]. A large-scale meta-analysis demonstrated that each 5 kg/m^2^ increase in BMI correlates with a 10–29% elevated risk of AF/AFL incidence [6]. Notably, the Framingham Heart Study revealed that obese individuals experienced up to a 50% increased risk of new-onset AF [7]. With the persistently expanding population of overweight and obese individuals, obesity-related AF/AFL is emerging as a major global health burden. Critically, as obesity represents a modifiable risk factor for AF/AFL, timely interventions could substantially reduce the associated public health burden [3]. Accordingly, current clinical practice guidelines explicitly recommend weight management interventions for AF/AFL patients with BMI > 27 kg/m2^2^ [2].

Although researchers have well-established the association between obesity and AF/AFL, existing studies have not yet fully elucidated the detailed epidemiological characteristics of the obesity-related AF/AFL burden. Significant gaps persist, particularly in systematic assessments of geographic variations, sex-specific heterogeneity, and temporal evolution patterns. To address this knowledge gap, our study utilized the Global Burden of Disease (GBD) database to comprehensively evaluate the mortality and disability burden (quantified as Years of Life Lost (YLLs) and Years Lived with Disability (YLDs)) attributable to high BMI-associated AF/AFL from 1990 to 2021. Specifically, we examined current disease burden patterns, temporal trends, and stratifications by region, sex, age, and Sociodemographic Index (SDI). We anticipate that these findings will provide an evidence-based foundation for developing targeted prevention and control strategies for obesity-related cardiac arrhythmias.

## Methods

### Data source

This study utilized data from the Global Burden of Disease (GBD) 2021 database (accessible at http://ghdx.healthdata.org/gbd-results-tool). The GBD 2021 study provides comprehensive epidemiological estimates for 369 diseases and injuries and 87 risk factors across 204 countries and territories from 1990 to 2021. Using a comparative risk assessment framework, the GBD methodology quantifies risk-attributable burden by comparing observed health outcomes with counterfactual scenarios based on theoretical minimum risk exposure levels or specific historical exposure distributions.

### Exposure and outcome definition

We defined high body mass index (HBMI) as BMI ≥ 25 kg/m^2^ in adults aged ≥ 20 years [8]. From the GBD 2021 database, we extracted the following HBMI-attributable atrial fibrillation and flutter (AF/AFL) burden metrics for 1990–2021:

1. Annual death counts
2. Disability-adjusted life years (DALYs), calculated as: *DALYs* = *Years of Life Lost (YLLs)* + *Years Lived with Disability (YLDs)*
3. Age-standardized rates (ASRs) for all metrics

All metrics were stratified by sex, age group, and geographical region.

Burden Metric Calculation:

1. Mortality rate: Deaths per 100,000 population
2. DALY rate: DALYs per 100,000 population
3. Age-standardized rates (ASRs): Calculated using the direct method weighted by the GBD standard population age structure. This standardization minimizes confounding from population age distribution differences, enabling valid temporal and cross-regional comparisons.

### Disparity analysis

We quantified global inequalities in HBMI-attributable AF/AFL burden (HAB) using Gini coefficients based on age-standardized mortality and DALY rates across nations (1990–2021). The Gini coefficient ranges from 0 (perfect equality) to 1 (maximal inequality), reflecting between-country disparities in disease burden distribution.

### Temporal trend analysis

Temporal trends in HAB ASRs were quantified using estimated annual percentage change (EAPC) with 95% confidence intervals (CIs):

1. *EAPC* > *0:* Significant increasing trend
2. *EAPC* < *0:* Significant decreasing trend

### Gender and Socioeconomic Gradient Analysis

To examine gender disparities across socioeconomic strata, we:

1. Stratified the 31-year data into three periods (1990–2000, 2001–2010, 2011–2021)
2. Within each Sociodemographic Index (SDI) quintile, compared age-standardized mortality and DALY rates between males and females across 5-year age groups using:
  1. Independent samples *t*-test (normally distributed data)
  2. Wilcoxon rank-sum test (non-normally distributed data)
  3. Defined statistical significance at α = 0.05 (two-tailed)

*Example workflow for age-standardized mortality rates (55–59 years, 1990–2000):*

1. Assessed normality using Shapiro–Wilk tests (α = 0.05)
2. Applied independent *t*-test with Levene’s homoscedasticity verification if both groups met normality assumptions
3. Implemented Wilcoxon rank-sum test otherwise

### Regional cluster analysis

We performed hierarchical clustering analysis with *z*-score standardization to classify 21 GBD regions based on temporal patterns (1990–2021) of:

1. Age-standardized mortality rates (ASMRs)
2. Age-standardized DALY rates (ASDRs) for HAB.

### Statistical software

All analyses and visualizations used R statistical software (version 4.3.3; R Foundation for Statistical Computing).

## Results

### Global HAB in 2021

**Fig. 1:**
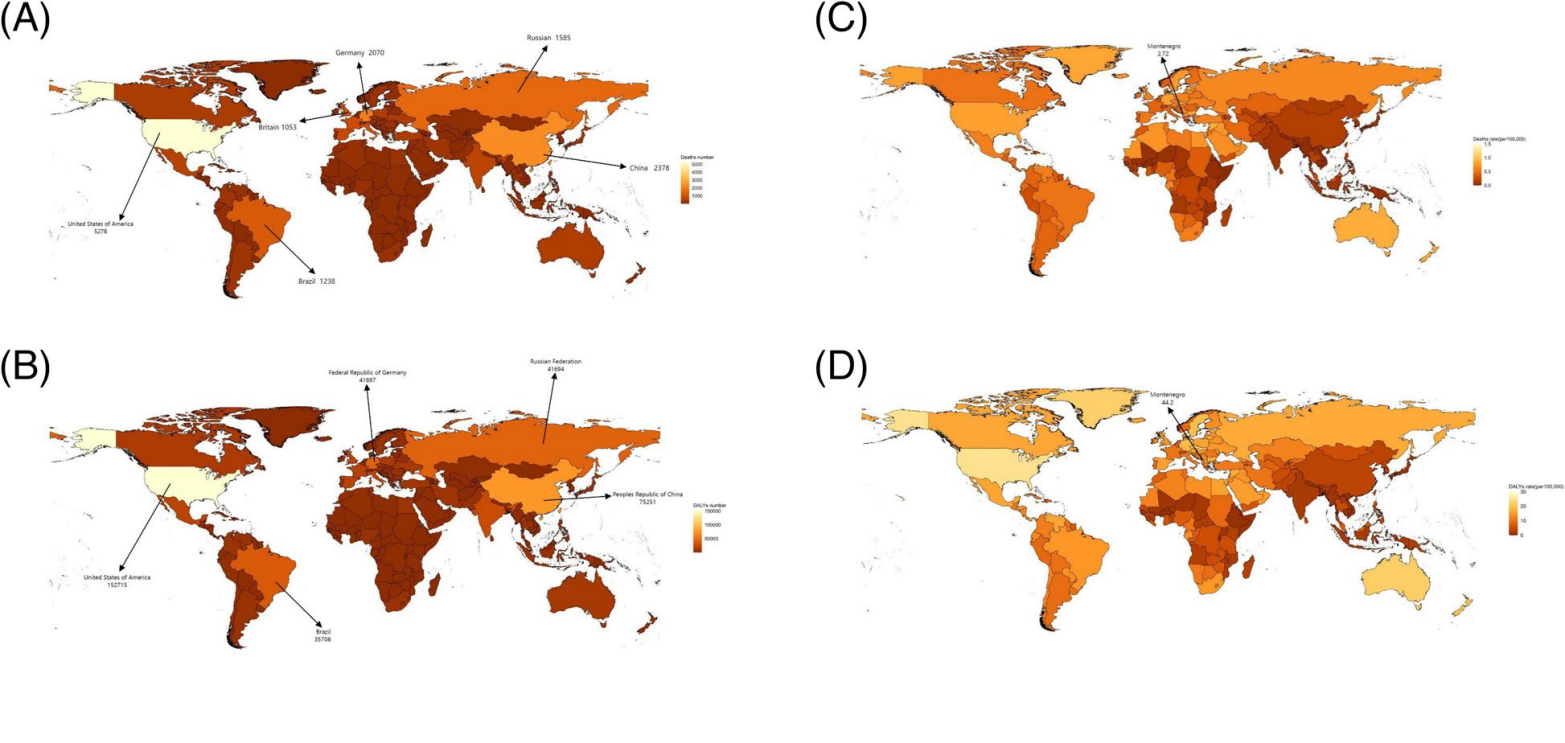
The global HBMI-related AF/AFL burden(HAB) in 2021 in 204 countries and territories. The absolute numbers (**A**&**B**) and age-standardized rates (per 100 000 persons) (**C**&**D**) of deaths and DALYs in 204 countries and territories. Abbreviations: AF/AFL, atrial fibrillation/atrial flutter; DALYs, disability-adjusted life years; HBMI, high body mass index; HAB, HBMI-related AF/AFL burden

In 2021, globally, HBMI-related AF/AFL caused approximately 27,236 deaths and 724,537 DALYs, representing increases of 376% and 314% since 1990, respectively. Globally, the age-standardized mortality and DALY rates reached 0.35 and 8.71 per 100,000 population, respectively, representing increases of 66.7% and 272.5% since 1990 (Supplementary Tables S1–S2). Notably, the United States had the highest absolute burden (5,278 deaths; 152,715 DALYs), followed by China, Germany, Russia, and Brazil (Fig. 1A, B). Countries with the highest rates clustered predominantly in Oceania, North America, and Western Europe, with Montenegro recording peak values for mortality (2.7/100,000) and DALY rates (44.1/100,000) (Fig. 1C, D; Supplementary Figure S1).

### Temporal trends in HAB

**Fig. 2:**
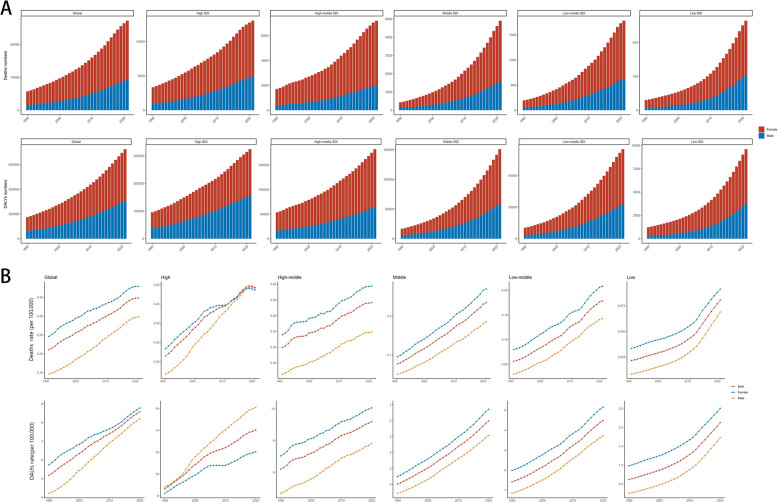
Global Burden of Atrial Fibrillation and/or Atrial Flutter Associated with High Body Mass Index by SDI Group, 1990–2021. **A** Trends in deaths and disability-adjusted life years (DALYs). **B** Trends in death and DALY rates

From 1990 to 2021, mortality and disability rates for high body mass index (HBMI)-associated atrial fibrillation and flutter (AF/AFL) showed spatially consistent increases globally. Notably, all regions demonstrated upward trends in both rates, as evidenced in Fig. 2A and B.

Deaths increased in all countries and regions, with East Asia, South Asia, and Southeast Asia experiencing rises exceeding 2000%. Similarly, HBMI-attributable DALYs showed increases > 1000% in these regions (Supplementary Fig. 2). Among the 21 GBD regions, age-standardized mortality rates (ASMRs) and DALY rates (ASDRs) demonstrated sustained increases over recent decades. Notably, South Asia showed the most pronounced mortality increases, with estimated annual percentage changes (EAPCs) of 8.07 (95% CI: 7.86–8.29) for ASMR and 7.1 (95% CI: 6.88–7.33) for ASDR (Figure S3).

Z-score hierarchical clustering analysis identified distinct geographic patterns in burden growth rates: while Europe, Australasia, and high-income North America experienced comparatively higher growth, East Asia, South Asia, Southeast Asia, and Sub-Saharan Africa (excluding South Sub-Saharan Africa) showed relatively lower increases (Supplementary Fig. 3).

### Differences among SDI quintiles

The global HBMI-attributable AF/AFL burden (HAB) varied substantially across Sociodemographic Index (SDI) quintiles. Over the past three decades, absolute HAB increased in all SDI quintiles. By 2021, the high-SDI quintile (deaths: 13,035; DALYs: 324,193) and high-middle-SDI quintile (deaths: 7,208; DALYs: 182,168) accounted for nearly three-quarters of the global burden, while the low-SDI quintile had the lowest burden (deaths: 264; DALYs: 9,595) (Supplementary Tables 1–2).

Mortality and DALY rates increased significantly across all quintiles, with the highest rates occurring in the high-SDI quintile (0.49 and 14.21 per 100,000, respectively). Notably, we observed a positive correlation between both mortality/DALY rates and SDI (Fig. 2B). Across 204 countries and territories, we identified a negative correlation between SDI and EAPC for both DALYs (r = −0.335; P < 0.001) and mortality (r = −0.228; P < 0.001; Pearson correlation).

However, regional deviations emerged: among 21 GBD regions, mortality and DALY rates fell below SDI-based predictions in East Asia, high-income Asia Pacific, South Asia, and southern Latin America (Supplementary Fig. 1).

### Gender and age heterogeneity

**Fig. 3:**
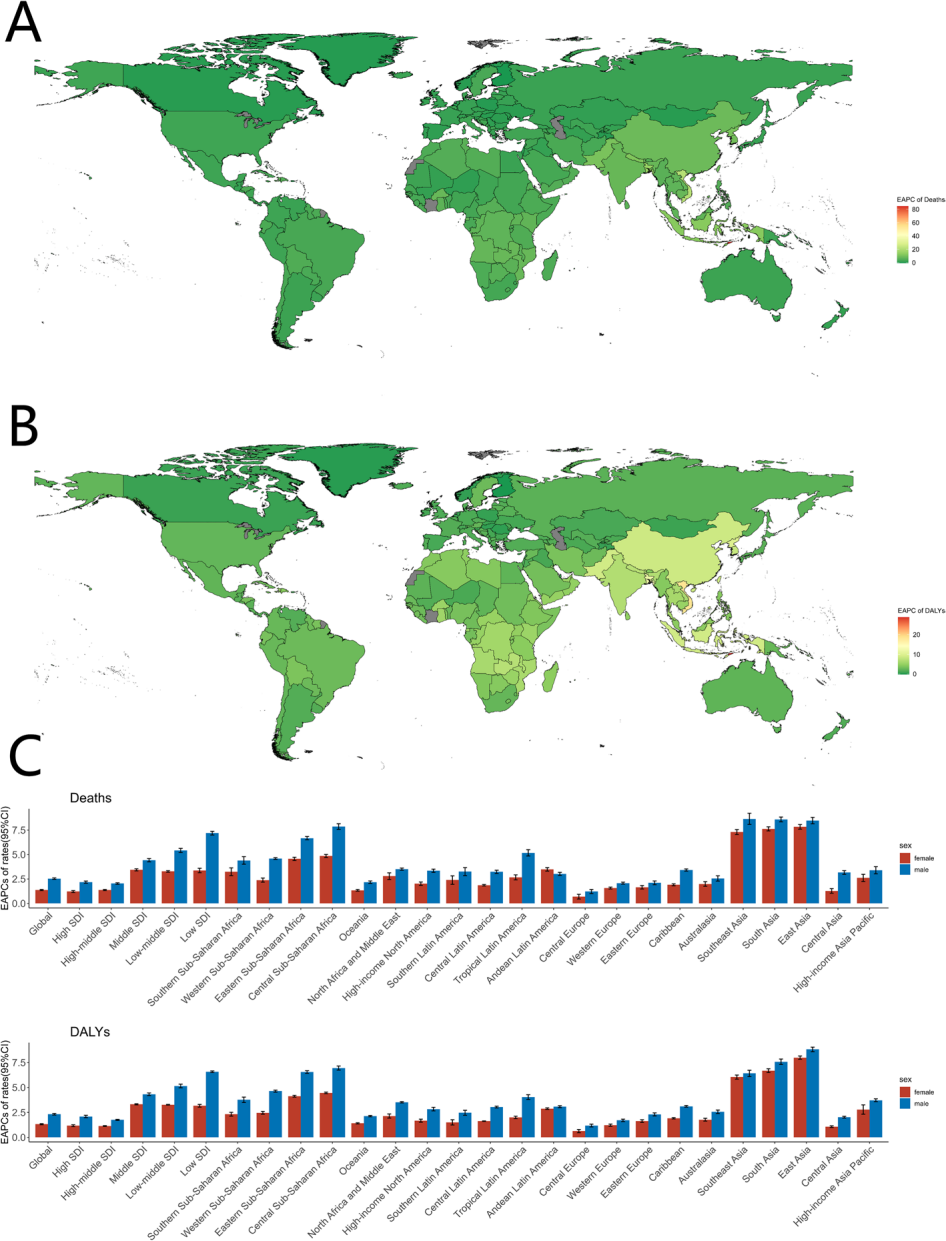
Estimated Annual Percentage Change (EAPC) in Mortality Rates and Disability-Adjusted Life Year (DALY) Rates for Atrial Fibrillation and/or Atrial Flutter Attributable to High Body Mass Index, 1990–2021. **A** EAPC in mortality rates across 204 countries. **B** EAPC in DALY rates across 204 countries. **C** EAPC in mortality and DALY rates by sex across 21 regions

From 1990 to 2021, significant differences in the HBMI-attributable AF/AFL burden (HAB) were observed between sexes (Figs. 2, 3C; Supplementary Figure S3; and Supplementary Figure S6). Globally, females had higher absolute numbers of deaths and DALYs than males, along with higher mortality rates and DALY rates. Among SDI quintiles, males exhibited higher mortality and DALY rates than females in the high-SDI quintile, whereas females maintained higher rates in all other SDI quintiles (Fig. 2B). Globally, mortality rates increased annually for both males and females, with male rates rising faster (Fig. 3C).

Hypothesis testing conducted on mortality and disability rates of high body mass index (HBMI)-related AF/AFL over the past 30 years (stratified by SDI groups) showed that—except for disability rates in high-SDI areas—mortality and disability rates in all other stratified groups consistently demonstrated a pattern of initial convergence with and subsequent exceeding of female rates (Supplementary Figure S4). Notably, when analyzed by age group, the estimated annual percentage change (EAPC) for both mortality and DALY rates in middle-aged/older adults (50–69 years) and older adults (≥ 70 years) maintained upward trends globally while also exhibiting inverse correlations with SDI (Supplementary Figure S5). Deaths and DALYs related to HBMI-associated AF/AFL increased with age, peaking at 90–94 years for mortality and 74–80 years for disability, followed by plateaus extending through 80–84 years (Supplementary Figure S6B).

### Contribution of HAB

According to the GBD 2021 study encompassing six major risk factors—elevated blood pressure, HBMI, high-sodium diet, alcohol use, smoking, and lead exposure—HBMI constituted the second-largest contributor to AF/AFL. Significant heterogeneity in risk factor profiles occurred across geographic locations, SDI quintiles, age groups, and sexes (Supplementary Figures S6-S7). Notably, the proportional HAB was highest in high-SDI regions (accounting for 19.8% of deaths and 20.9% of DALYs) versus lowest in low-SDI regions (5.8% of deaths; 6.8% of DALYs) (Supplementary Figure S6A). Among 21 GBD regions, North Africa and the Middle East had the highest proportional burden (28.4% of deaths; 29.1% of DALYs), while high-income Asia Pacific had the lowest (4.7% of deaths; 5.1% of DALYs) (Supplementary Figure S6B). Critically, all regions demonstrated substantial increases in HAB from 1990 to 2021 regardless of stratification criteria. Furthermore, although the proportional burden increased in both sexes during this period, males exhibited a more substantial elevation (Supplementary Figure S7), indicating sex-specific acceleration of obesity-related atrial cardiopathology.

### Global health inequality of HAB

Next, we calculated the Gini coefficient to assess global inequalities in HBMI-attributable AF/AFL burden (HAB). This analysis revealed improved geographic health equity across countries from 1990 to 2021 (Supplementary Figure S8). Specifically, the Gini coefficient for age-standardized mortality rates declined from 0.48 (1990) to 0.38 (2021), while the coefficient for DALY rates decreased from 0.45 to 0.35 during this period.

## Discussion

Paralleling the global surge in atrial fibrillation incidence [1]. Currently, both the prevalence of HBMI and the HAB have increased dramatically. The management of HBMI and obesity-associated cardiovascular diseases has thus become an increasingly significant challenge. The correlation between HBMI and AF/AFL burden may stem from shared risk factors, pathological mechanisms, and comorbidities linking obesity and AF. Specifically, obese patients exhibit excessive adipose tissue, which secretes adipokines, activates the renin-angiotensin system, and enhances oxidative stress. These processes trigger inflammatory responses, promote the migration and proliferation of smooth muscle cells, and impair endothelial function, thereby promoting the onset of cardiovascular diseases such as AF and CAD. These conditions mutually reinforce each other, forming a vicious cycle that collectively accelerates heart failure, stroke, and cardiovascular death, thereby increasing the burden associated with AF/AFL [2]. Furthermore, obesity-related expansion of epicardial adipose tissue is considered a key driver of AF, mediated through paracrine signaling and direct tissue infiltration [3]. A prospective cohort study on AF demonstrated that overweight and obesity were associated with an increased risk of major adverse events and mortality in AF patients [4]. However, some studies have reported a contradictory phenomenon—termed the "obesity paradox"—in which overweight and obese AF patients exhibit a reduced risk of cardiovascular and all-cause mortality [5]. A possible explanation is that overweight and obese patients typically receive earlier, more intensive treatment and closer follow-up monitoring, while obesity itself may provide greater metabolic reserve. Currently, it is of critical importance to comprehensively elucidate the HAB, including its sex- and region-specific disparities.

This study provides a comprehensive assessment of the HAB. The findings reveal that over the past three decades, HBMI-related AF/AFL has contributed to a significant increase in absolute deaths, DALYs, and age-standardized mortality and morbidity rates worldwide, with notable heterogeneity across regions, sexes, and temporal trends.

Across all SDI regions, ASDRs and ASMRs demonstrated consistent increases. Notably, the EAPC of ASDRs and ASMRs showed an inverse relationship with SDI (Supplementary Fig. 3 C), while absolute ASMRs and ASDRs values exhibited a positive correlation with SDI (Supplementary Fig. 1). This trend remained consistent when analyzed across bifurcated age groups (Supplementary Fig. 5). The HAB was highest in high-SDI regions, which accounted for 45% of the global AF/AFL burden in 2019 – representing a decline from 1990 levels yet still substantially exceeding the proportions observed in low- and middle-SDI regions. These findings align with the conclusions reported by Tan et al [6]. These observed patterns may be explained by the following factors: First, obesity-driven HBMI affects disease burden across all age groups as a prevalent metabolic disorder [7]. Second, the rapidly increasing obesity prevalence in low- and middle-SDI countries has accelerated the growth rate of HAB, thereby narrowing the inter-SDI disparity over time. Third, populations in high-SDI regions face greater obesity risks due to socioeconomic advantages that paradoxically facilitate unhealthy lifestyles—including tobacco use, alcohol consumption, and high-fat diets. This has led to particularly severe (and still worsening) obesity epidemics in developed nations, as exemplified by the extraordinary obesity rates documented in the United States [9]. Furthermore, existing studies indicate that elevated BMI is associated with an increased risk of cardiovascular disease events [8]. Thereby contributing to a greater burden of AF/AFL.

Notably, regions including East Asia, High-income Asia Pacific, South Asia, and Southern Latin America—despite their high economic development—exhibited lower mortality and disability rates without demonstrating the expected SDI-correlated disease burden and growth trends (Fig. 3C, Supplementary Fig. 1). This phenomenon may be attributed to ethnic, dietary, cultural, educational, and healthcare resource factors. For instance: Stronger weight-management motivation among Japanese women due to prevailing aesthetic standards [10]. Predilection for fish, shellfish, and plant-based diets in Japan and Korea-a dietary pattern significantly associated with reduced all-cause and cardiovascular mortality risks [11]. Lau et al. reported a higher prevalence of atrial fibrillation in Caucasian populations compared to Asia–Pacific nations such as China and Japan [12]. Although Australia, as part of the high-income Asia–Pacific region, exhibited elevated mortality and disability rates, its relatively small population size minimized its impact on aggregate regional data [13]. These region-specific determinants are not readily translatable to other settings for effective disease burden management. The pathogenesis of obesity involves complex multifactorial mechanisms, with economic development demonstrating a strong epidemiological association with obesity prevalence [14]. As economic growth continues to drive the obesity epidemic, regions with currently moderate SDI indices may inevitably experience disease burdens comparable to high-SDI regions without implementing comprehensive preventive strategies. Regrettably, current evidence indicates that despite widespread implementation of national weight-control interventions, no country has achieved sustained reversal of obesity trends, with the exception of temporary improvements observed during economic recessions.

Existing studies have demonstrated that the Gini coefficient serves not only as a measure of regional income inequality but also as a valuable tool for comparing disparities in disease burden across regions [15]. Our analysis reveals a consistent decline in the Gini coefficient for global HAB from 1990 to 2021 (Supplementary Fig. 8), suggesting progressive improvement in health inequality among nations. However, given the methodological constraints inherent in Gini coefficient calculations, the precise magnitude of this health inequality reduction remains undetermined.

Regarding gender disparities, to mitigate potential confounding by cultural, regional, and economic factors, we conducted stratified analyses by region and SDI level when comparing mortality and disability rates between sexes. The results consistently demonstrated significantly higher rates in women across nearly all subgroups (Supplementary Figs. 9A-9B). Similarly, the HAB was markedly greater in women—a pattern readily explained by their longer life expectancy (Supplementary Figs. 9C-9D) [16]. Current evidence further indicates that HBMI exerts a greater impact on AF disease burden in women than in men [17, 18]. This disparity may be attributed to both higher prevalence and greater severity of obesity among females [7]. In 2021, the prevalence of obesity among women was 20.8%, significantly higher than the 14.8% observed in men. Furthermore, Blüher et al. reported that severe obesity (BMI > 40 kg/m^2^) was 2.5 times more prevalent in women (1.6%) compared to men (0.64%) [19]. Furthermore, influenced by sex hormones and gut microbiota diversity, body composition exhibits significant heterogeneity between genders. At comparable BMI levels, women typically demonstrate lower muscle mass proportion and greater propensity for visceral adipose tissue accumulation [20, 21]. Women exhibit a higher prevalence of sarcopenic obesity and demonstrate greater susceptibility to the adverse cardiac functional impacts of central obesity compared to men [22]. Mechanistically, these effects may be mediated through sympathetic nervous system activation, pro-inflammatory responses, mitochondrial dysfunction, oxidative stress generation, and dysregulation of the renin–angiotensin–aldosterone system (RAAS). These pathways collectively contribute to vascular endothelial dysfunction, cardiomyocyte injury, and myocardial fibrosis [23, 24]. Potentially explaining why HBMI contributes disproportionately to cardiovascular disease incidence and AF/AFL burden in women.

A unique gender disparity pattern was observed in high-SDI regions, where male disability rates have consistently exceeded female rates since 1990—a trend contrasting with both other regions and mortality patterns within high-SDI areas (Fig. 2B). Age-stratified analysis (Supplementary Fig. 4) revealed an anomalous progressive trend: the age threshold at which male disability rates surpassed female rates shifted from 70–74 years (1990–1999) to 75–79 years (2000–2009), and further to 80–84 years (2010–2021). This phenomenon may be attributed to two synergistic factors: male AF patients demonstrate lower mortality risk [25], and obesity prevalence in high-SDI regions has undergone a gender crossover, with male rates now exceeding female rates [7].

The risk factor analysis revealed a substantial increase in the proportion of HAB among males since 1990, with the attributable fraction becoming significantly higher than in females (Supplementary Fig. 7). These findings suggest that over the past three decades, certain sex-specific factors have amplified the detrimental impact of elevated BMI more prominently in males compared to females. The accelerated progression in both prevalence and severity of obesity among male populations may represent a key explanatory factor for these observed epidemiological shifts [19].

AF/AFL is an age-related disorder, and the GBD data demonstrate that the AF/AFL burden attributable to obesity is disproportionately greater in older populations—a finding consistent with established pathophysiological mechanisms (Supplementary Figure S6B). Concurrently, current epidemiological data reveal a marked increase in obesity prevalence among young adults over recent decades [25]. This trend suggests that the full impact of contemporary obesity rates on future disease burden has not yet been realized. From a preventive perspective, targeted interventions including enhanced screening protocols and health education campaigns for this demographic should be prioritized to mitigate the anticipated surge in HAB.

Furthermore, it is noteworthy that AF induced by HBMI is not an isolated health issue. HBMI serves as a significant pathogenic factor for both AF and coronary artery disease (CAD) by inducing inflammation, oxidative stress, and endothelial dysfunction [26]. Simultaneously, CAD, being a major complication of AF, exhibits an intricate link with AF, forming a vicious cycle where each disease promotes the other.

Epidemiologically, extensive studies confirm that individuals with either AF or CAD are more susceptible to developing the other condition. Specifically, 18–40% of patients diagnosed with AF also have CAD [27]. At the molecular level, inflammation, oxidative stress, and endothelial dysfunction represent shared pathogenic mechanisms. AF contributes to endothelial dysfunction, which itself is an early manifestation of atherosclerosis [28]. AF promotes CAD by inducing endothelial dysfunction. Furthermore, CAD, often presenting as myocardial ischemia, exacerbates all three mechanisms underlying the initiation and perpetuation of AF: focal ectopic firing, re-entry, and neural remodeling [29]. Given that heart disease resulting from CAD is a leading cause of death globally, imposing a substantial healthcare burden and severely impairing public health [30], these findings underscore that controlling the epidemic trend of HBMI-induced AF offers substantial benefits that necessitates urgent intervention.

### Study limitations

The potential influence of economic, healthcare, and cultural factors on GBD data sources remains incompletely quantified. While our use of SDI-stratified gender comparisons has substantially minimized potential confounding, residual biases may persist due to unmeasured regional variations in disease ascertainment and reporting practices. Second, Detailed information on different AF/AFL subtypes (such as paroxysmal, persistent, or permanent) cannot be obtained from the GBD database, which may limit the comprehensive and stratified analysis of the HAB. Third, similar to the classification of atrial fibrillation subtypes, the current analysis did not further stratify HBMI categories. Given variations in obesity prevalence and severity across nations and regions, the included populations may demonstrate significant heterogeneity between different geographical areas. While BMI remains a convenient and widely used screening tool for overweight and obesity, this metric fails to differentiate between fat mass distribution patterns. Consequently, BMI proves inadequate for characterizing geriatric obesity, abdominal adiposity, and visceral fat accumulation – all of which exhibit distinct metabolic consequences. Fourth,notably, GBD estimates are derived from modeled data rather than direct clinical measurements, potentially limiting their accuracy in certain national and regional contexts, particularly in settings with constrained surveillance infrastructur,furthermore, it is not possible to infer direct causality between HBMI and AF/AFL from these trend data.Future real-world studies are warranted to more precisely characterize disease trends and burden, particularly in low- and middle-SDI regions where data representativeness may be compromised. Fifth,

## Conclusion

Our analysis focused on identifying epidemiological trends between high body mass index (HBMI) and atrial fibrillation/flutter (AF/AFL) rather than establishing causal relationships. This yielded three key findings:Disparities in the HBMI-attributable AF/AFL burden (HAB) have narrowed significantly across sociodemographic strata over the past three decades, reflecting progressive mitigation of structural health inequities.First,economic advancement propagates the HAB across the development spectrum, a trajectory confirmed by our temporal analysis.second,the current burden profile observed in high-SDI regions suggests the future epidemiological path for lower-SDI settings.Third,consequently, targeted screening and early therapeutic intervention in young obese cohorts may mitigate future burden escalation among aging populations.Furthermore, the reasons underlying the greater absolute burden of HBMI-related AF/AFL among males in high-SDI regions require further exploration. One key limitation of this investigation is the absence of atrial fibrillation subtype stratification, which affects the generalizability of our findings to specific clinical subpopulations. Future studies should therefore integrate detailed phenotyping protocols aligned with contemporary clinical AF/AFL management guidelines.

## Supplementary Information

Supplementary Material 1

Supplementary Material 2

## Data Availability

The data supporting this study are openly available from the Global Burden of Disease (GBD) database.
