# Supplementary material for: Global burden of atrial fibrillation/flutter attributable to a high body mass index (HBMI) from 1990–2021
Source: BMC Cardiovasc Disord. 2025 Oct 14;25:741. doi: 10.1186/s12872-025-05125-5 (PMC12522503; doi:10.1186/s12872-025-05125-5)
Supplement: Supplementary file 1 — Supplementary Material 1 [file 12872_2025_5125_MOESM1_ESM.doc]

**Supplementary Material for:**Global Burden of Atrial Fibrillation/Flutter Due to High Body Mass Index from 1990 to 2021: Estimates from the Global Burden of Disease Study 2021

**Supplementary Figure S1.** AF/AFL Burden Attributable to HBMI (2021): By Location and SDI Quintile. [2](#__RefHeading___Toc203848289)

**Supplementary Figure S2.** Temporal Change in HBMI-Attributable AF/AFL Burden: 204 Countries/Regions. [3](#__RefHeading___Toc203848290)

**Supplementary Figure S3.** Clustered Heatmap: Deaths/DALYs Rates (HBMI-Attributable AF/AFL) by GBD Region, 1990–2021. [4](#__RefHeading___Toc203848291)

**Supplementary** **Figure 4.** Decadal Trends in Sex-Stratified Disability and Mortality Rates . [5](#__RefHeading___Toc203848292)

**Supplementary Figure S5.** EAPCs of Deaths/DALYs Rates (1990–2021): By Age Group, Sex, and SDI . [6](#__RefHeading___Toc203848293)

**Supplementary Figure S6** Proportion of AF/AFL Risk Factors: by SDI and GBD Region [8](#__RefHeading___Toc203848294)

**Supplementary Figure S7.** Proportion of AF/AFL Deaths/DALYs Attributable to Risk Factors: By Sex . [8](#__RefHeading___Toc203848295)

**Supplementary** **Figure 8.** Gini Coefficients: HSBP-Attributable AF/AFL Burden (1990–2021) [9](#__RefHeading___Toc203848296)

**Supplementary Figure S9.** Age/Sex Distribution of HBMI-Attributable AF/AFL Burden: 1990 vs. 2021 [10](#__RefHeading___Toc203848297)

**Supplementary Figure S10.** HBMI-Attributable AF/AFL Burden (2021): By Sex and Country/Region . [11](#__RefHeading___Toc203848298)

[**Supplementary TABLE 1/2** HBMI-Attributable AF/AFL Burden: Deaths/DALYs Cases, Age-Standardized Rates, and Temporal Trends (1990–2021) 12](#__RefHeading___Toc203851615)


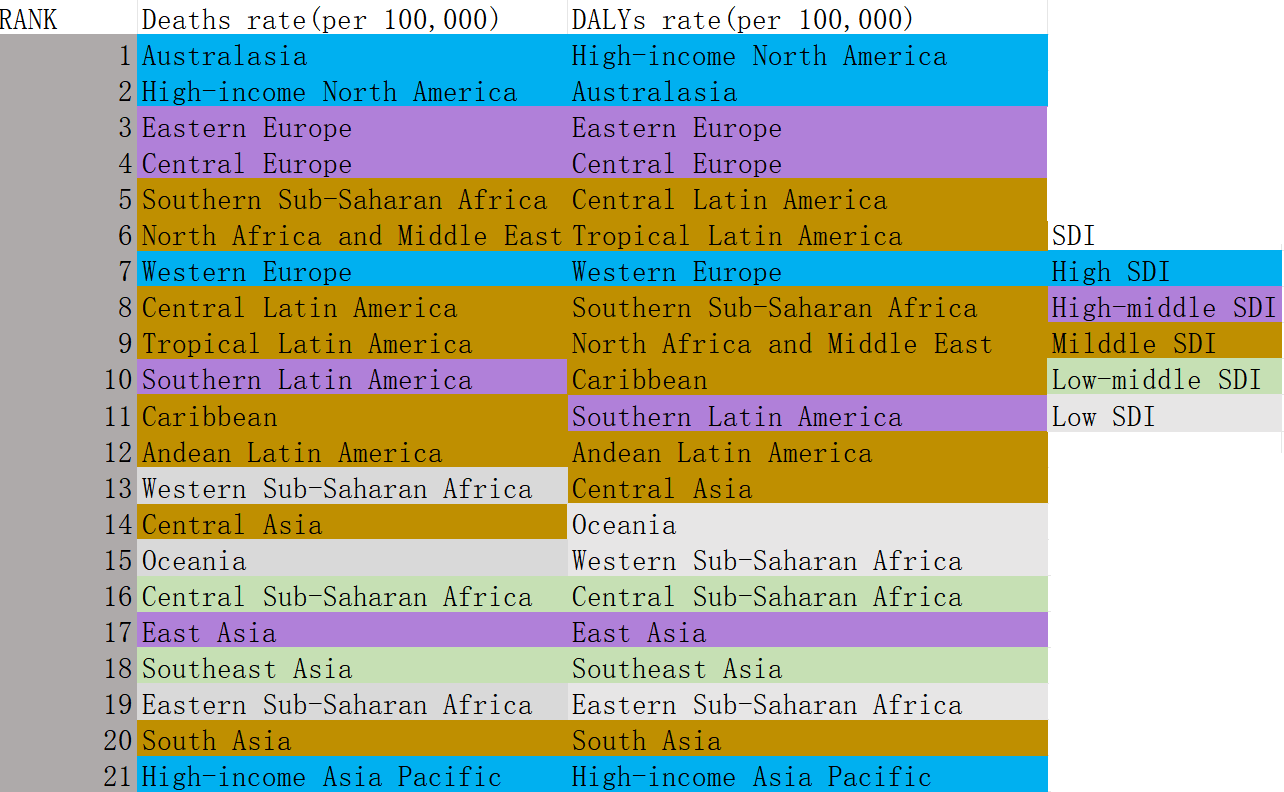


**Supplementary Figure S1.** Global burden of atrial fibrillation/atrial flutter (AF/AFL) attributable to high body mass index (HBMI) in 2021, by location and Socio-Demographic Index (SDI) quintile. Ranking of deaths and DALYs (per 100,000 population) across the 21 Global Burden of Disease (GBD) regions, which were divided into five groups based on their SDI: low SDI (0-0.455), low-middle SDI (0.456-0.608), middle SDI (0.609-0.690), high-middle SDI (0.690-0.805), and high SDI (0.806-1). HBMI, high body mass index; AF/AFL, atrial fibrillation/atrial flutter; DALYs, disability-adjusted life years; GBD, Global Burden of Disease; SDI, Socio-Demographic Index.


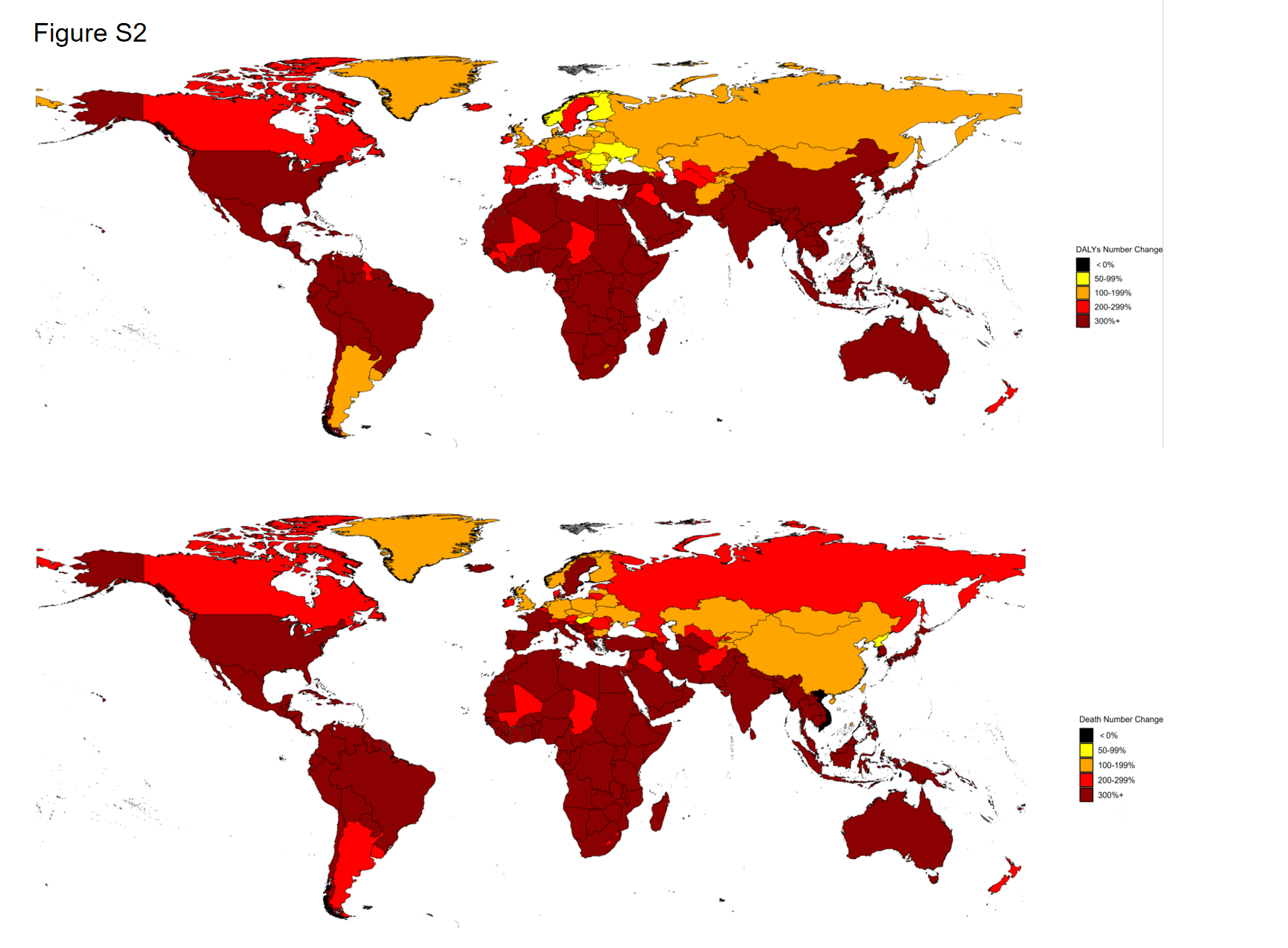


**Supplementary Figure S2.** Changes in the burden of atrial fibrillation/atrial flutter (AF/AFL) attributable to high body mass index (HBMI) across 204 countries and regions. Percentage changes in the number of deaths (A) and DALYs (B) across 204 countries and regions from 1990 to 2021. Abbreviations are the same as in Figure S1.


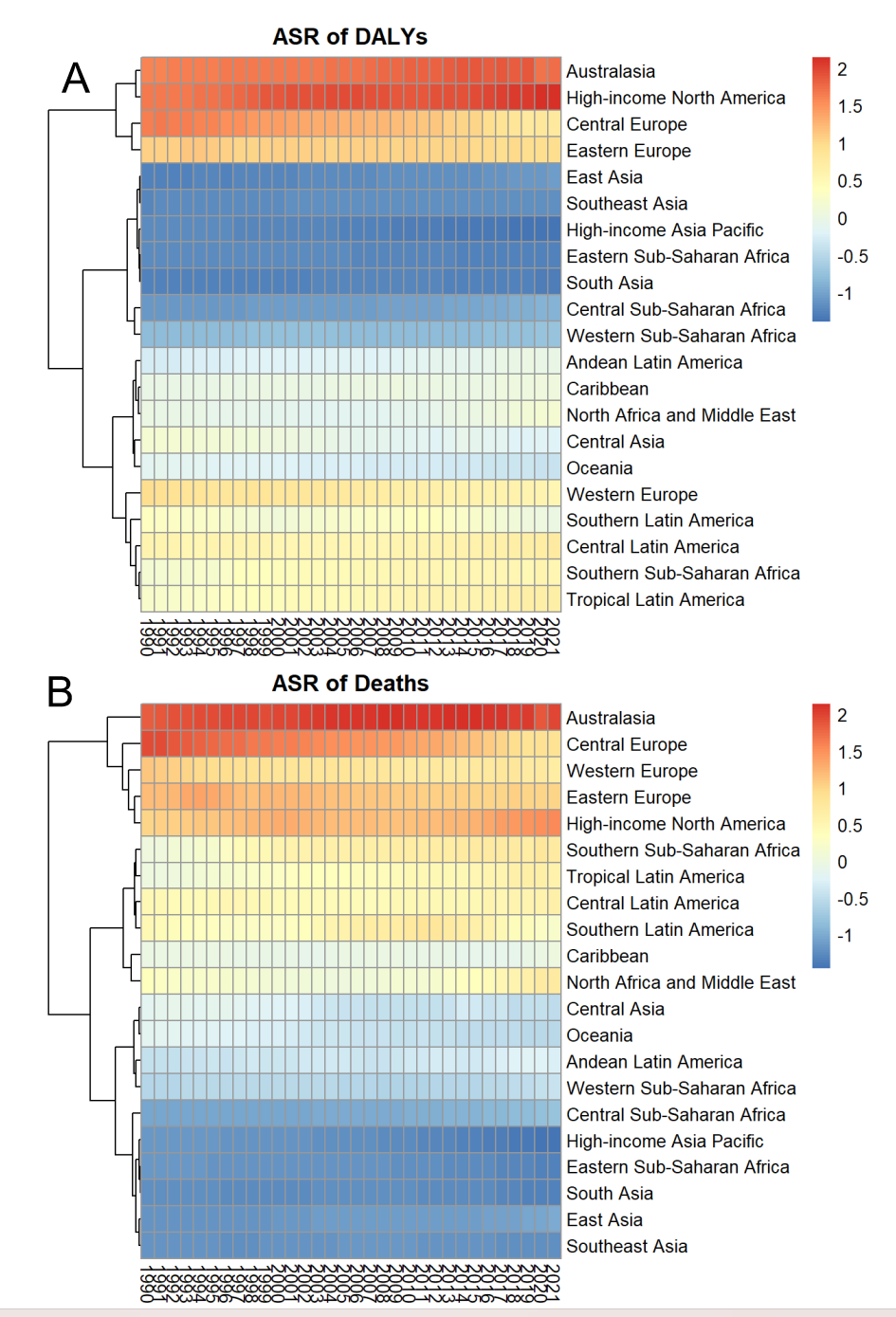


**Supplementary Figure S3.** Clustered heatmap of standardized rates (per 100,000 population) of deaths and DALYs attributable to HBMI for AF/AFL across the 21 Global Burden of Disease (GBD) regions, along with temporal trends from 1990 to 2021.(A) Standardized rates of DALYs attributable to HBMI for AF/AFL;(B) Standardized rates of deaths attributable to HBMI for AF/AFL.The z-scores of the standardized rates are based on squared Euclidean distance measures.ASR: Age-standardized rate; other abbreviations are the same as in Figure S1.

(A)





(B)


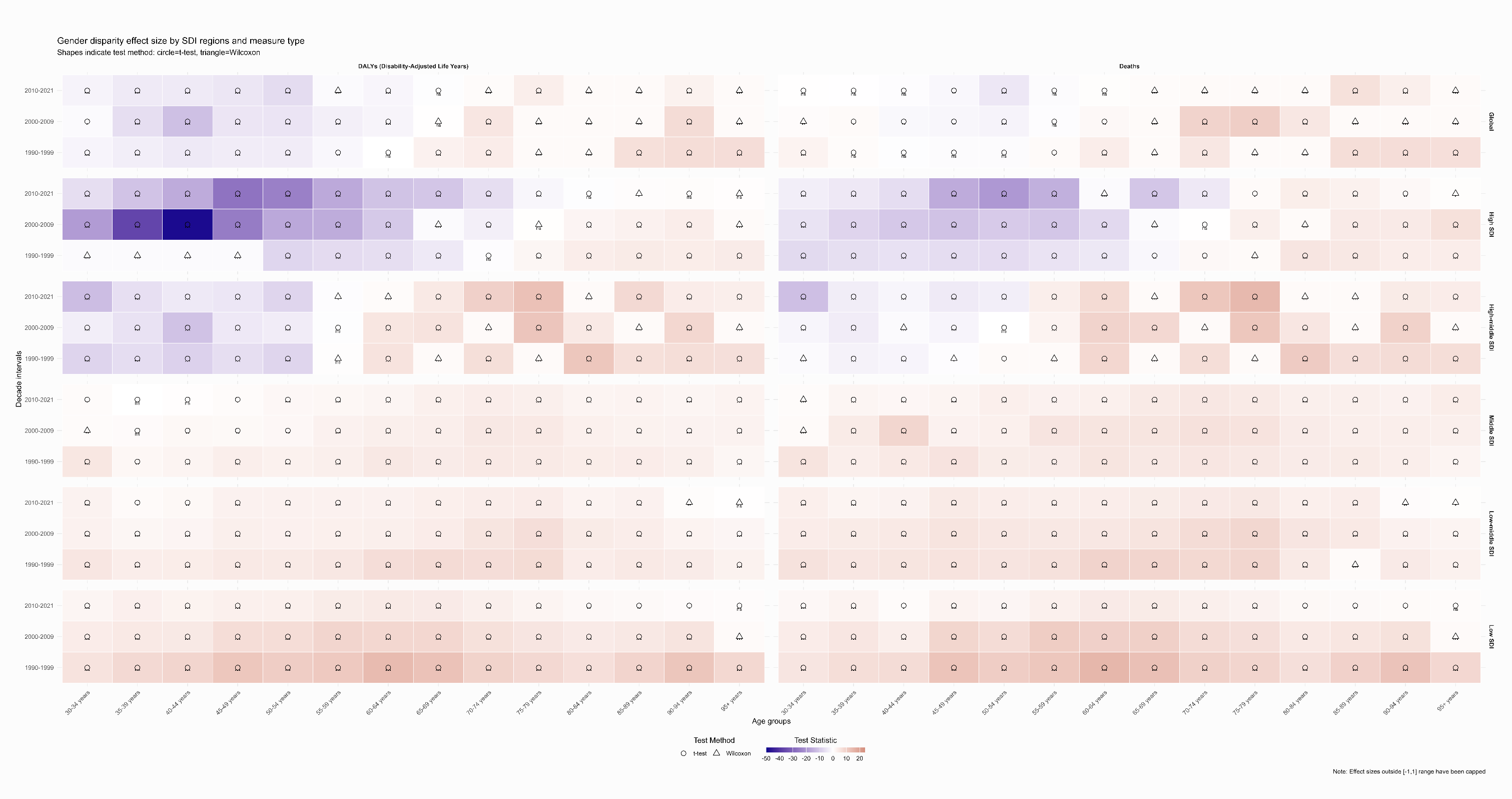


**Supplementary** **Figure 4. Longitudinal disability and mortality rates (stratified by sex) were aggregated into per-decade intervals for hypothesis testing of temporal trends. A: Test statistic constrained to [-1, 1] (e.g., correlation coefficient)B: Unconstrained estimation**





**Supplementary Figure S5.** EAPCs of deaths and DALYs rates for three age groups, by sex and SDI, 1990-2021.

EAPCs of deaths and DALYs rates for bot, male and femal in global and 5 SDI quintiles, 50-69 years, and 70+ years groups. EAPC, estimated annual percentage change; SDI, socio-demographic index; other abbreviations as in Figure S1.

(A)


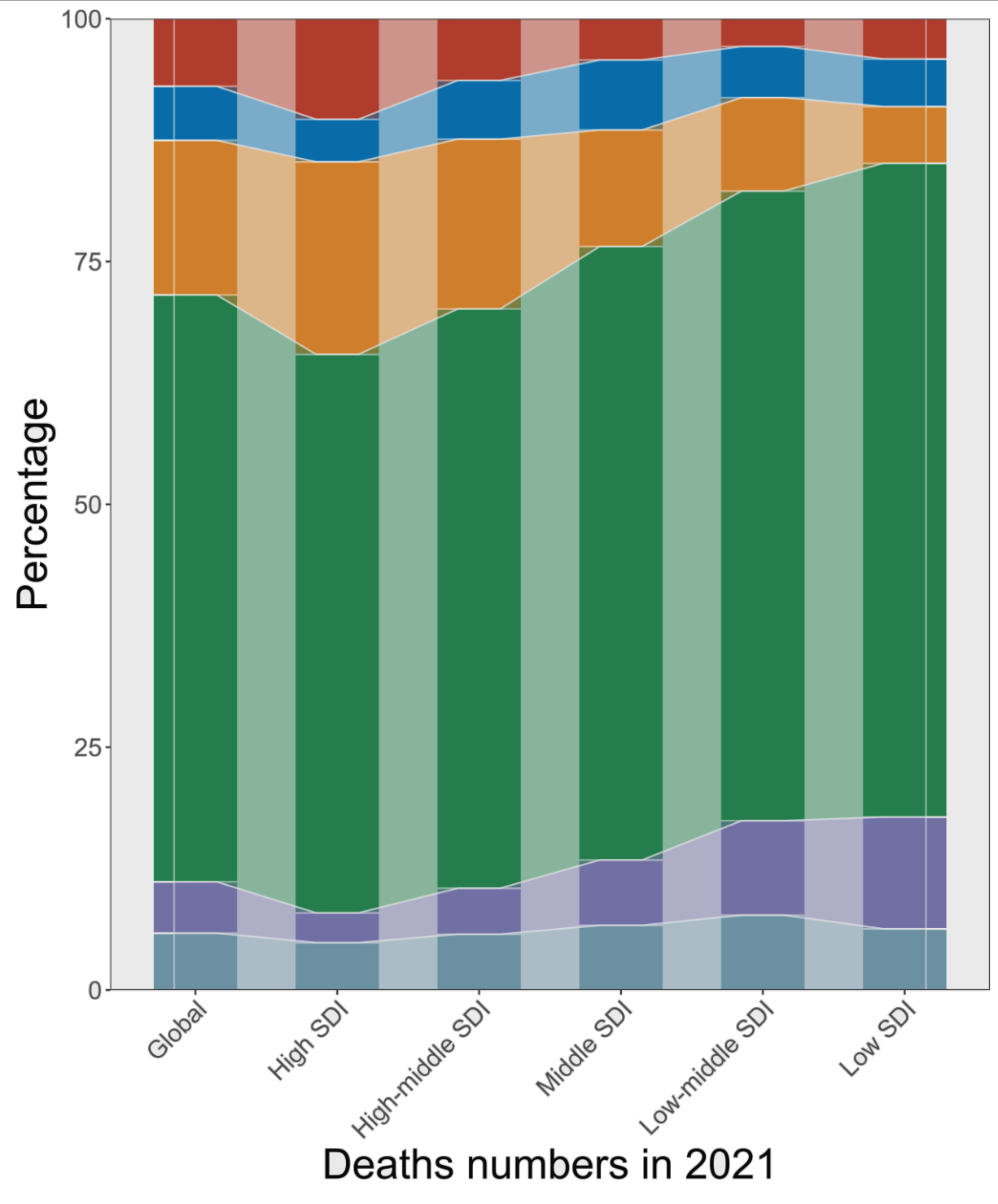

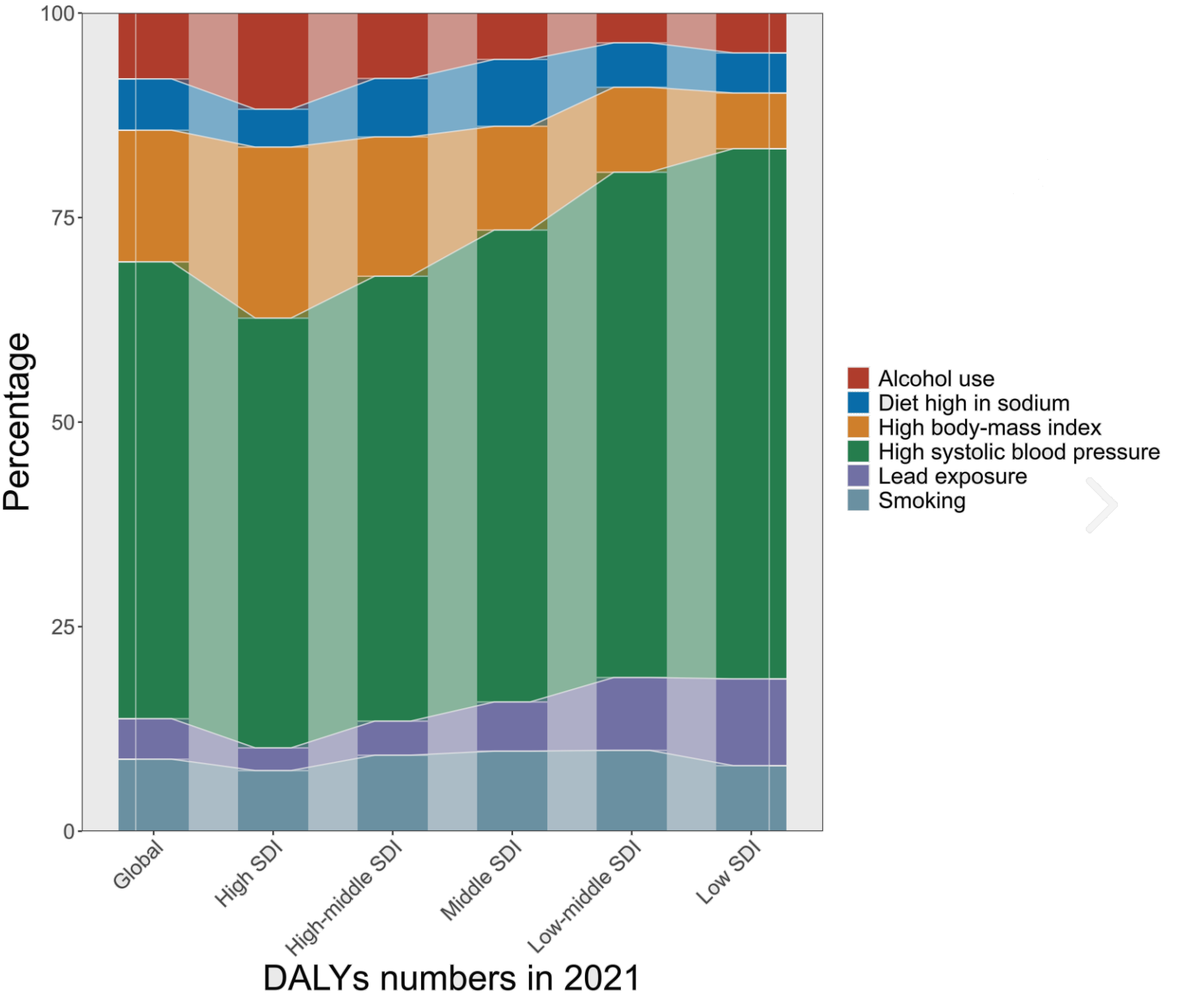


(B)


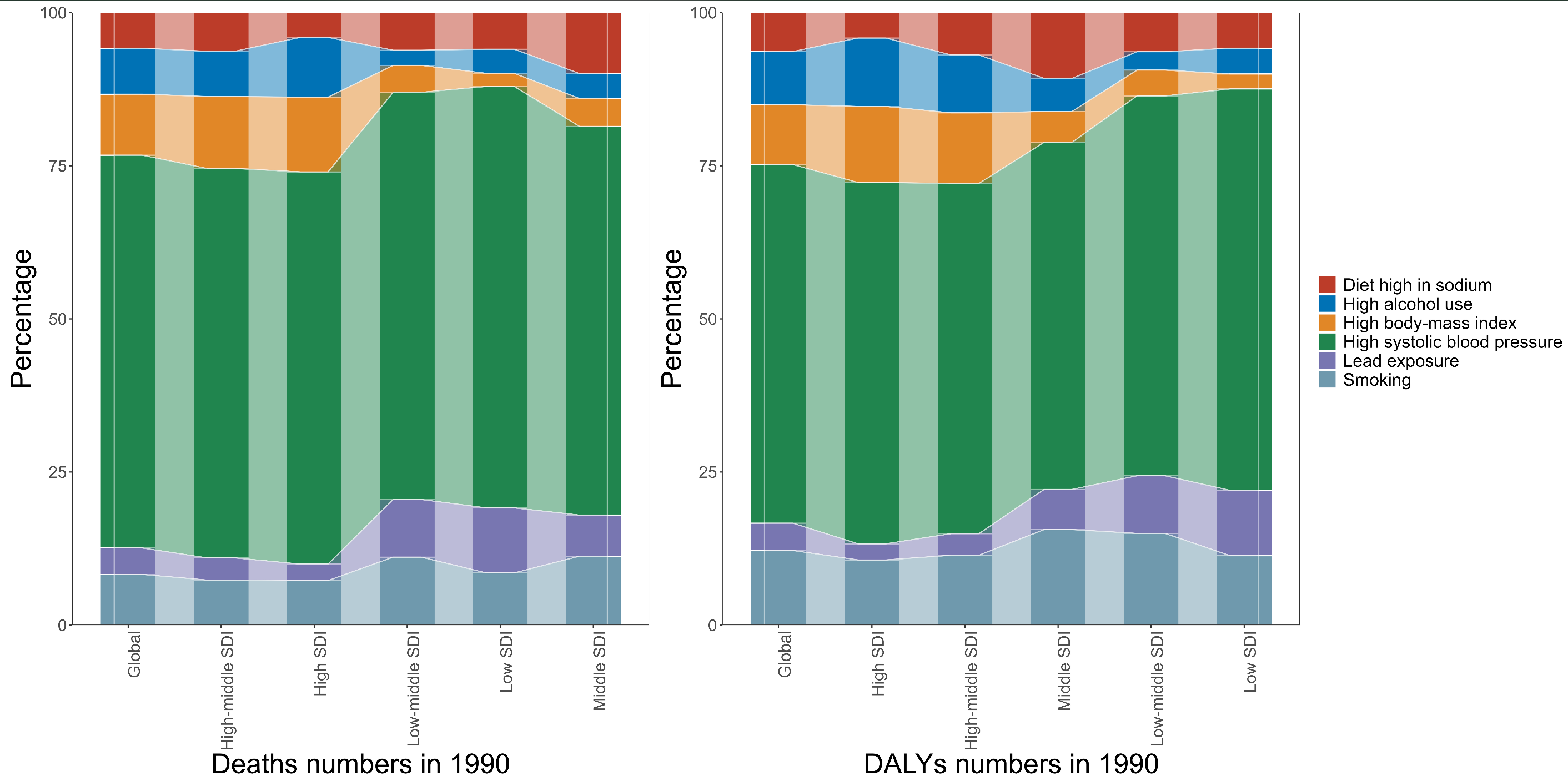

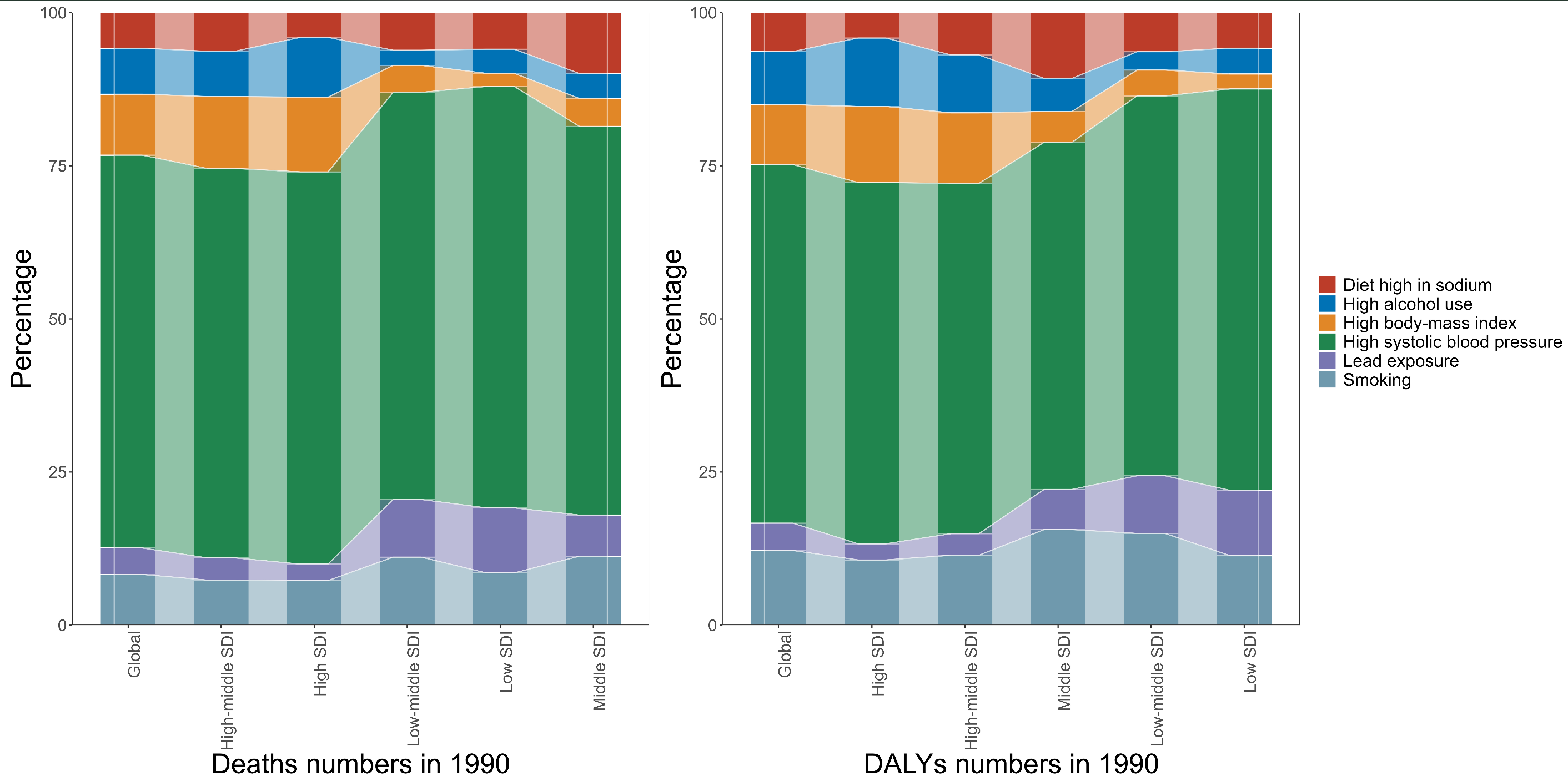


(C)


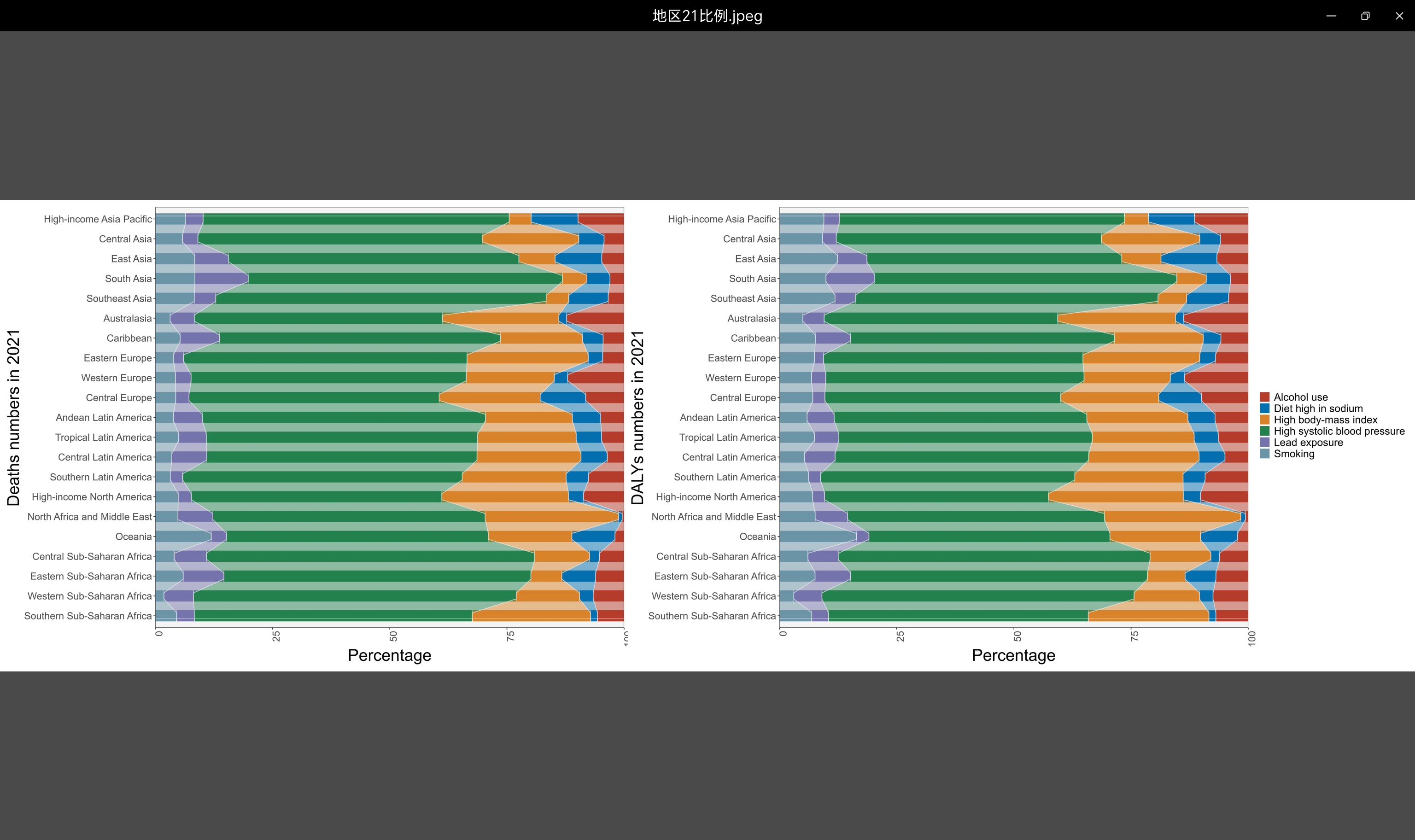


(D)


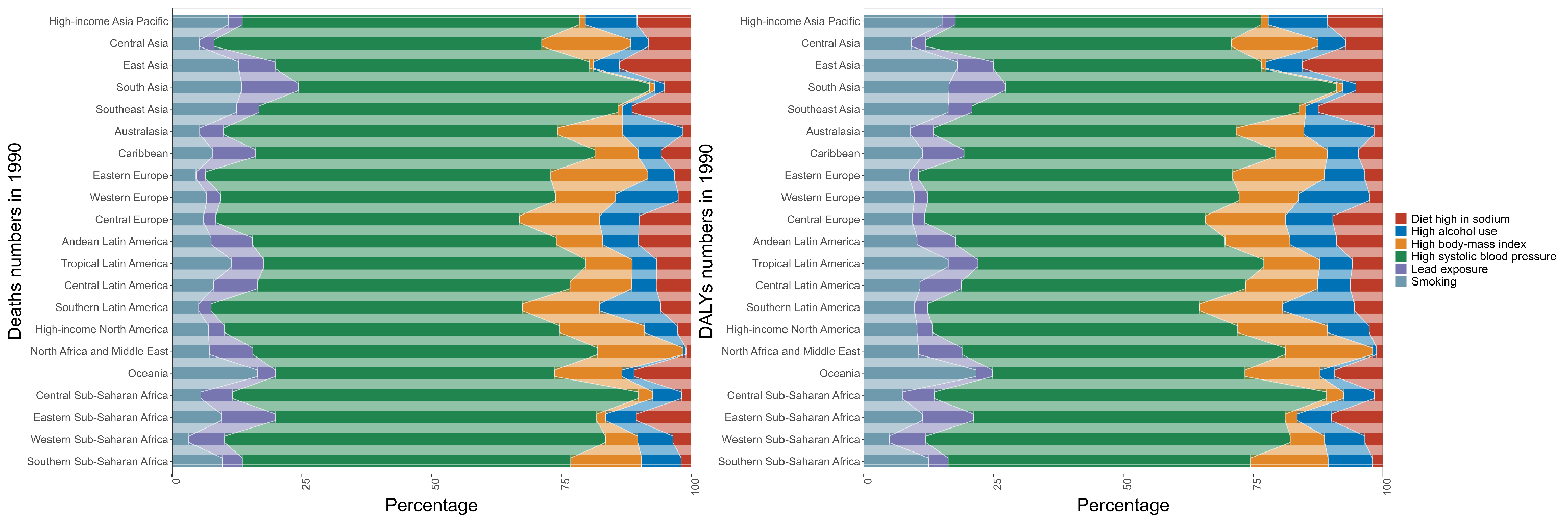


**Supplementary Figure S6** The proportion of risk factors contributing to AF/AFL, by SDI (A) and 21 GBD world regions (C) , in 2021. The proportion of risk factors contributing to AF/AFL, by SDI (B) and 21 GBD world regions (D) , in 1990.

1. (B)
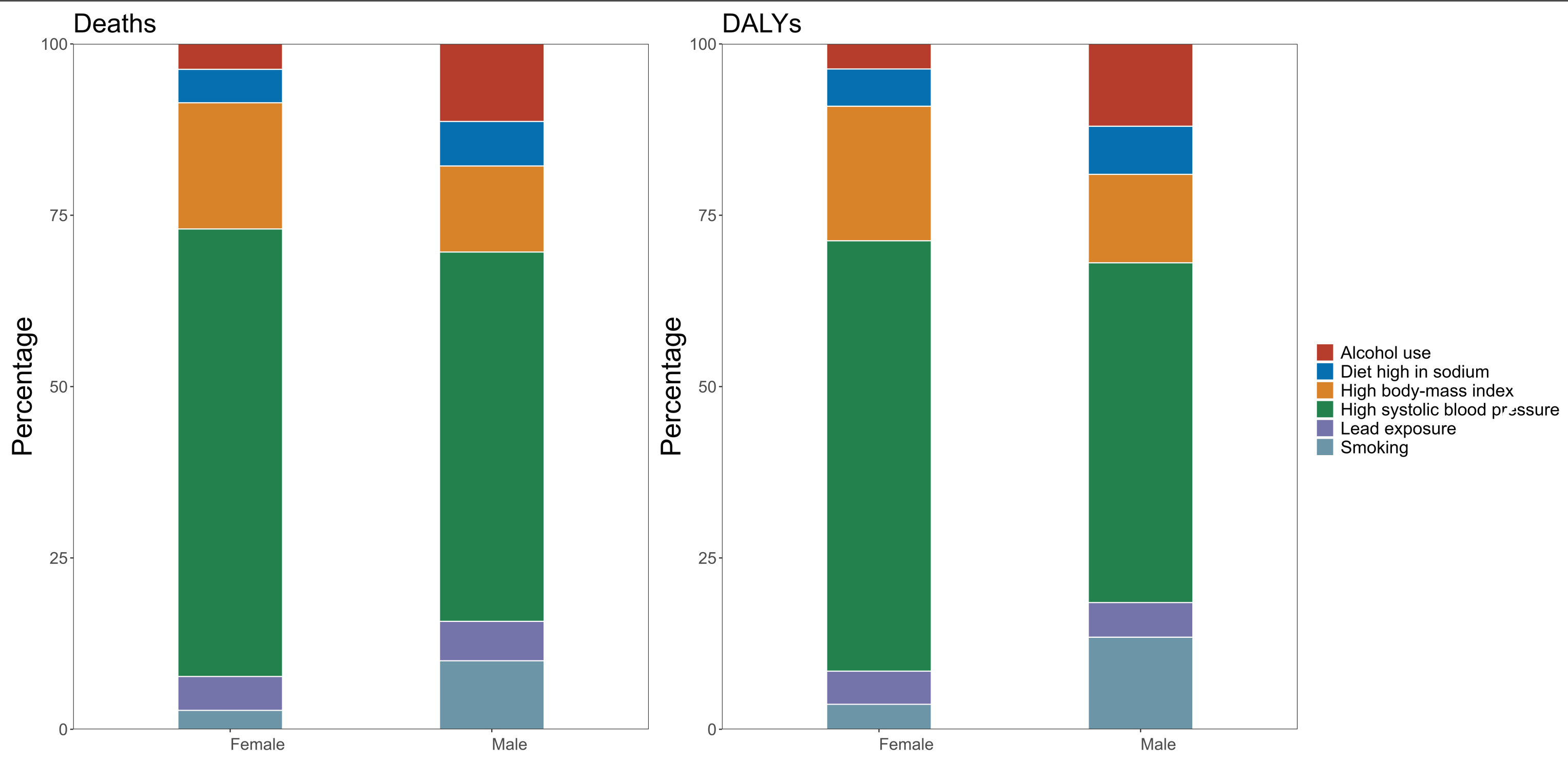


（C） （D）


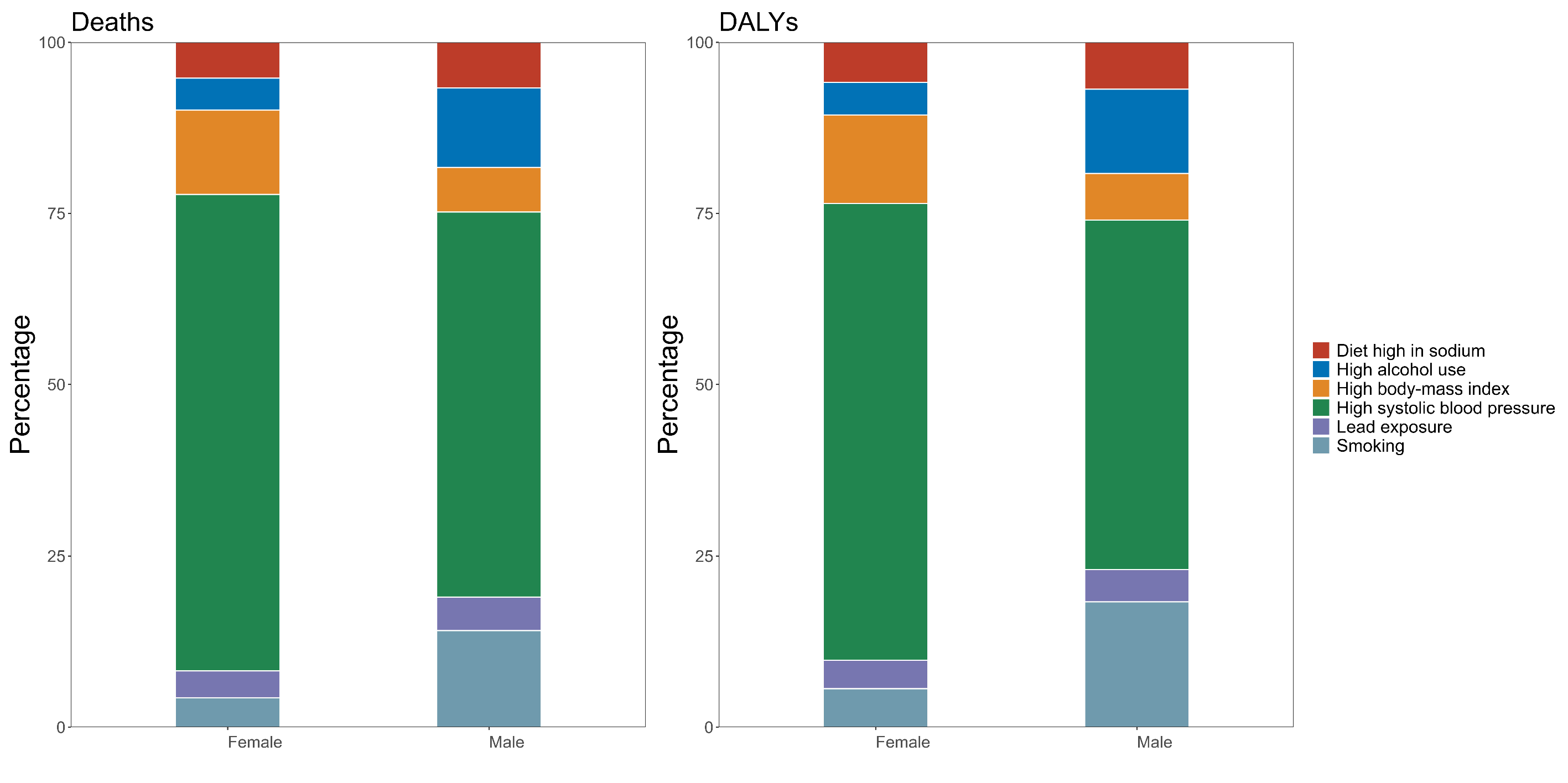


**Supplementary Figure S7.**The proportion of risk factors contributing to AF/AFL deaths and DALYs, by sex in 2021(A&B) and1990(C&D) .Abbreviations as in Figure S1.

(A) (B)


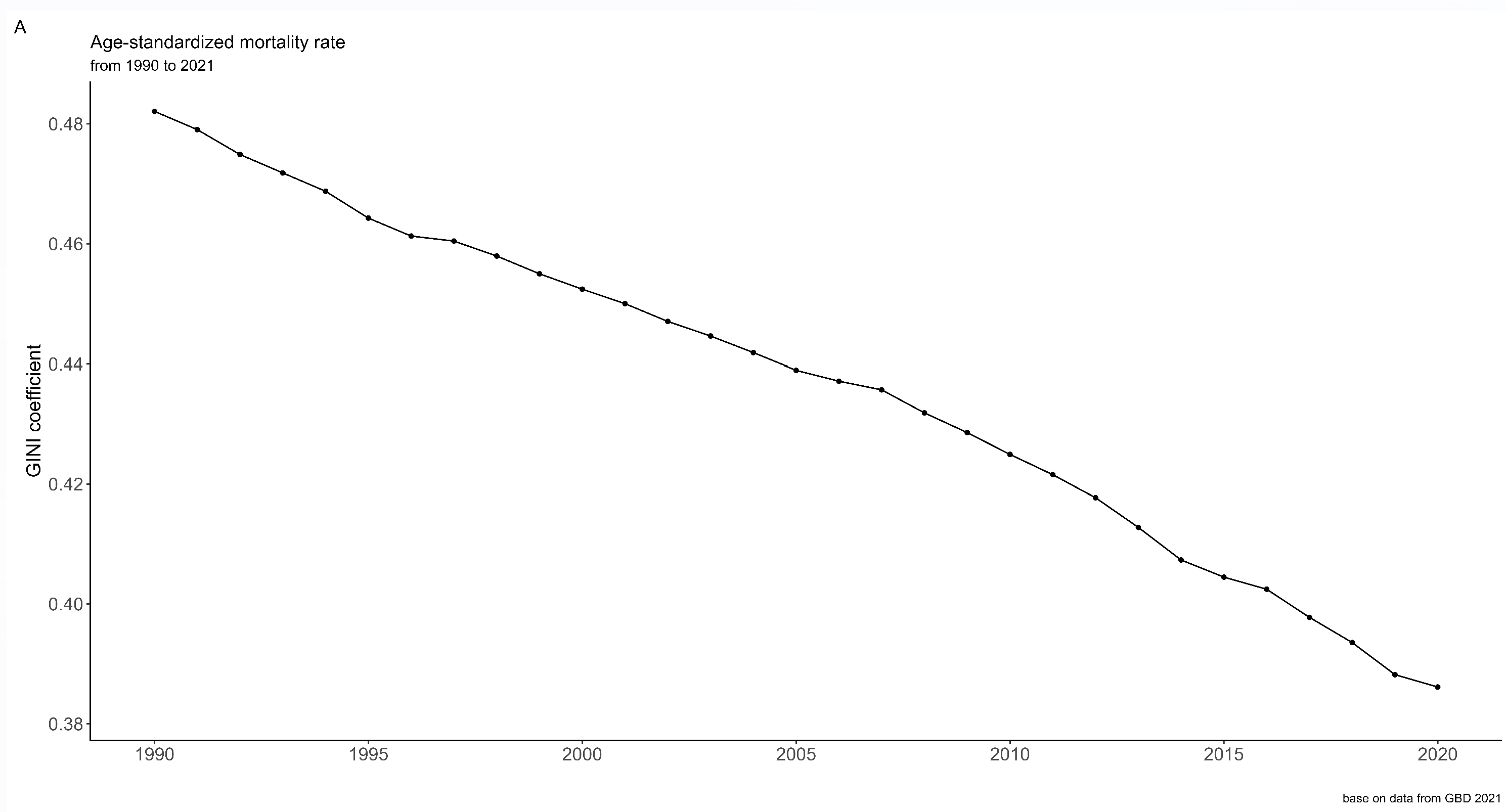

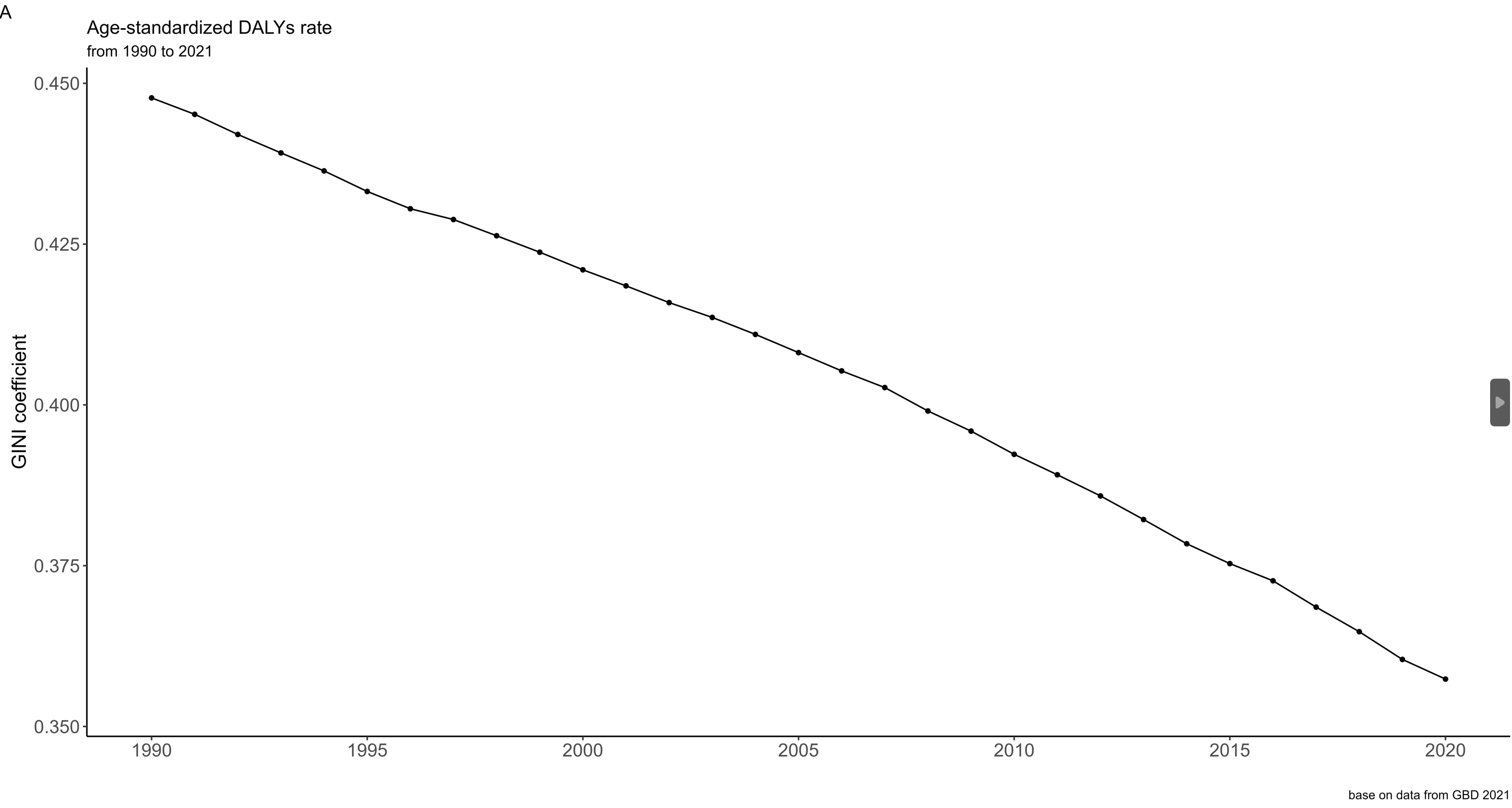


**Supplementary** **Figure 8.** The Gini coefficients of HSBP-related AF/AFL burden during 1990–2021. Trends in the Gini coefficients calculated based on (A) age-standardized deaths rates, (A) age-standardized DALYs rates across 204 countries and territories globally between 1990 and 2019.

(A)
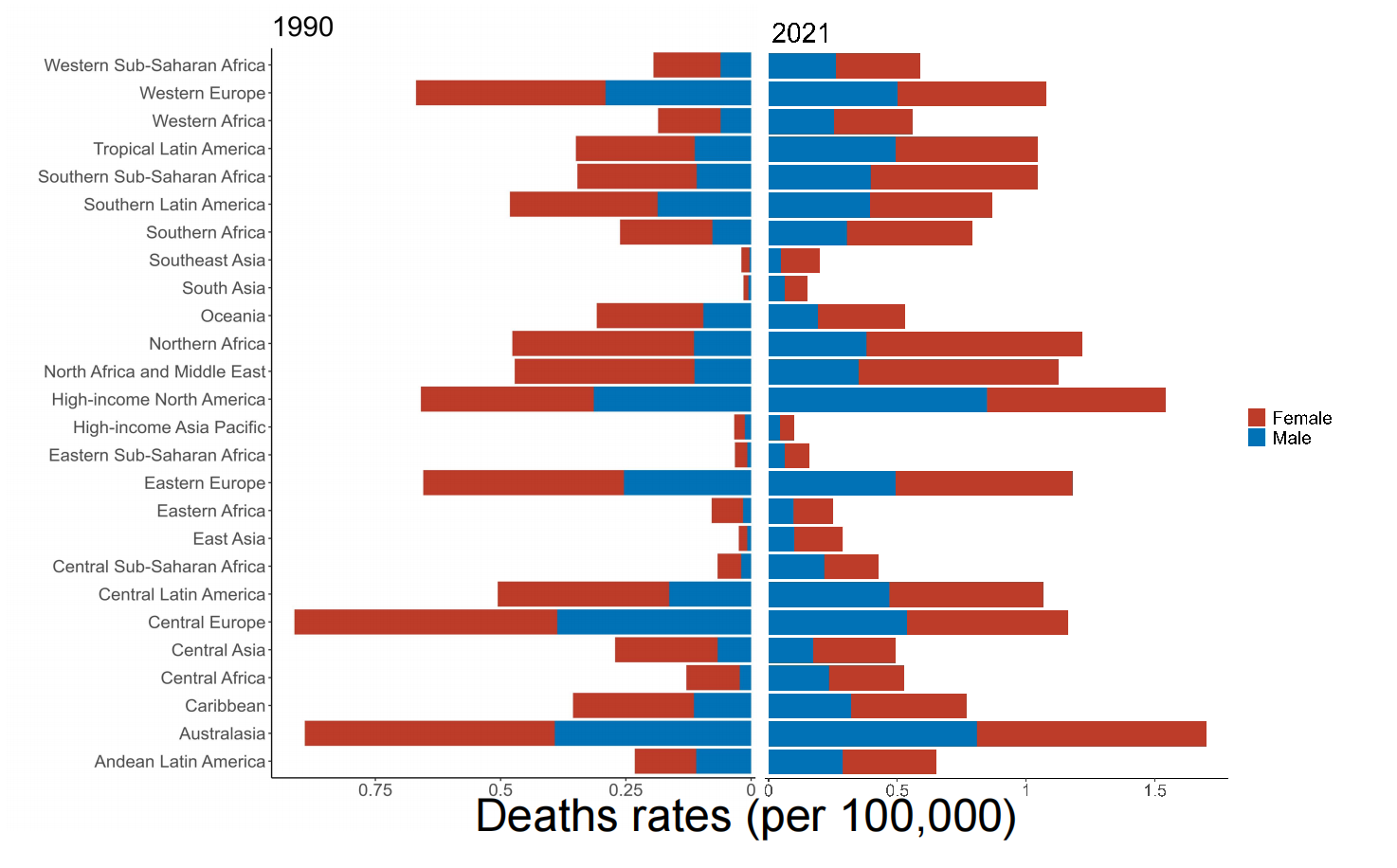

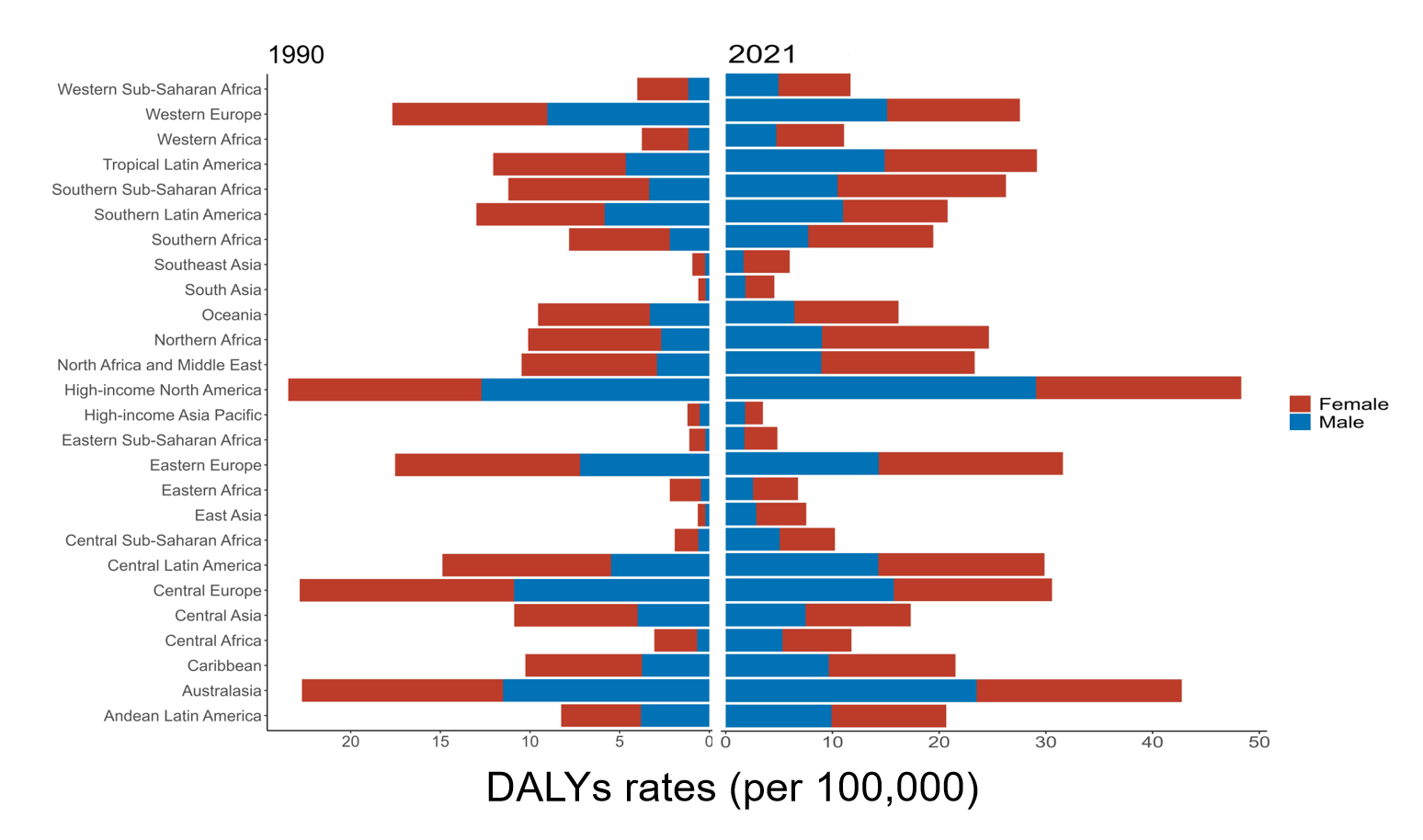


(B)


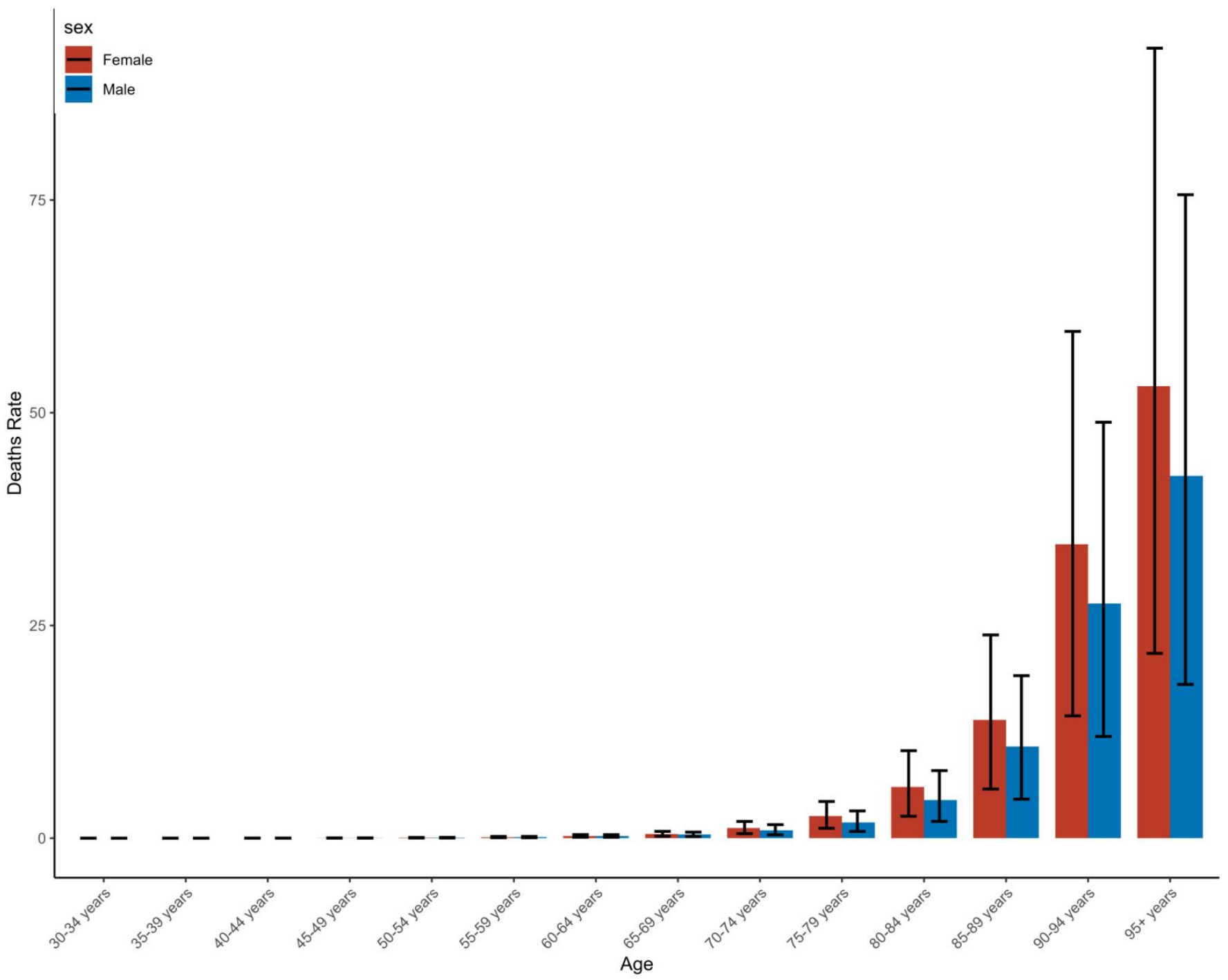

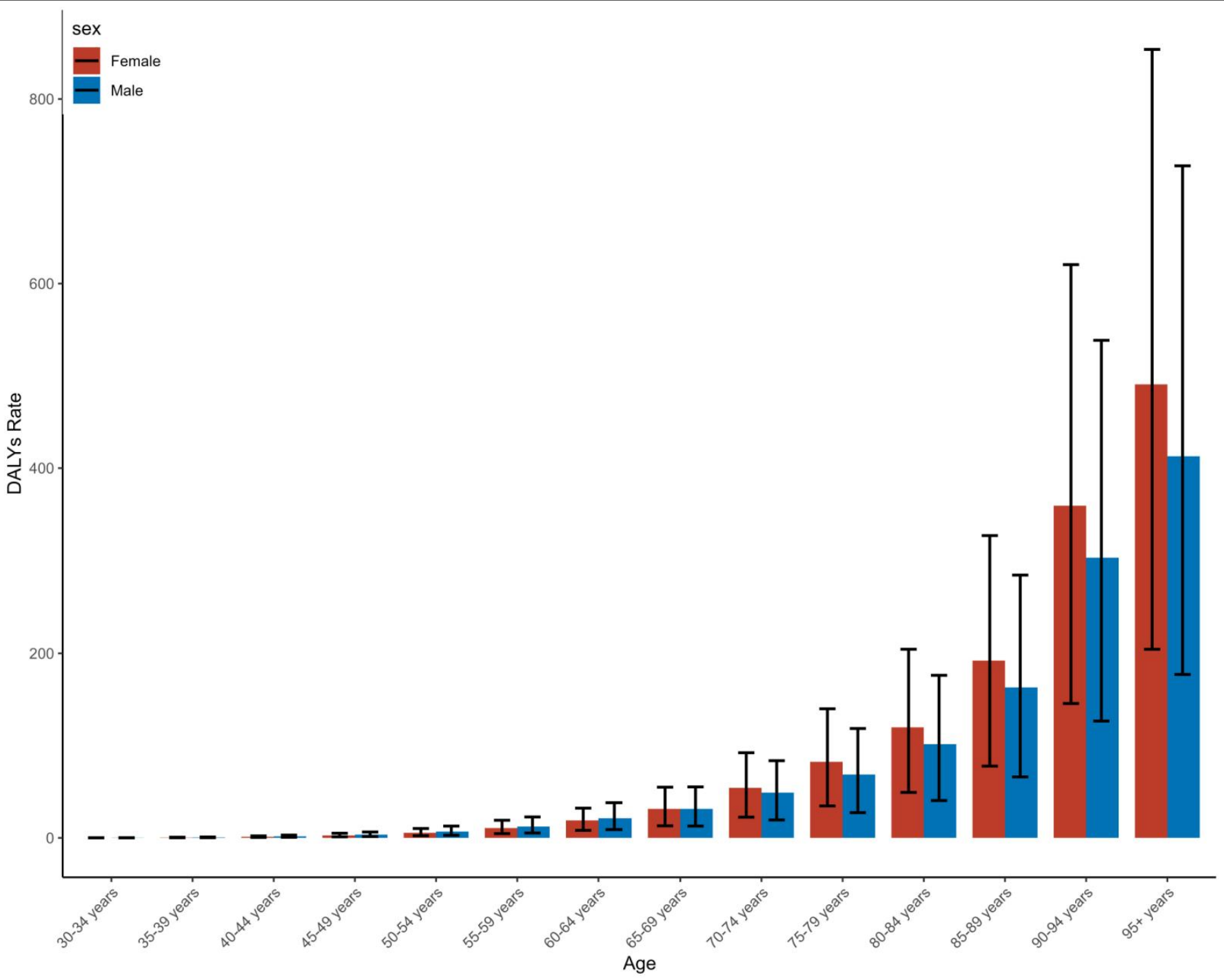


(C)


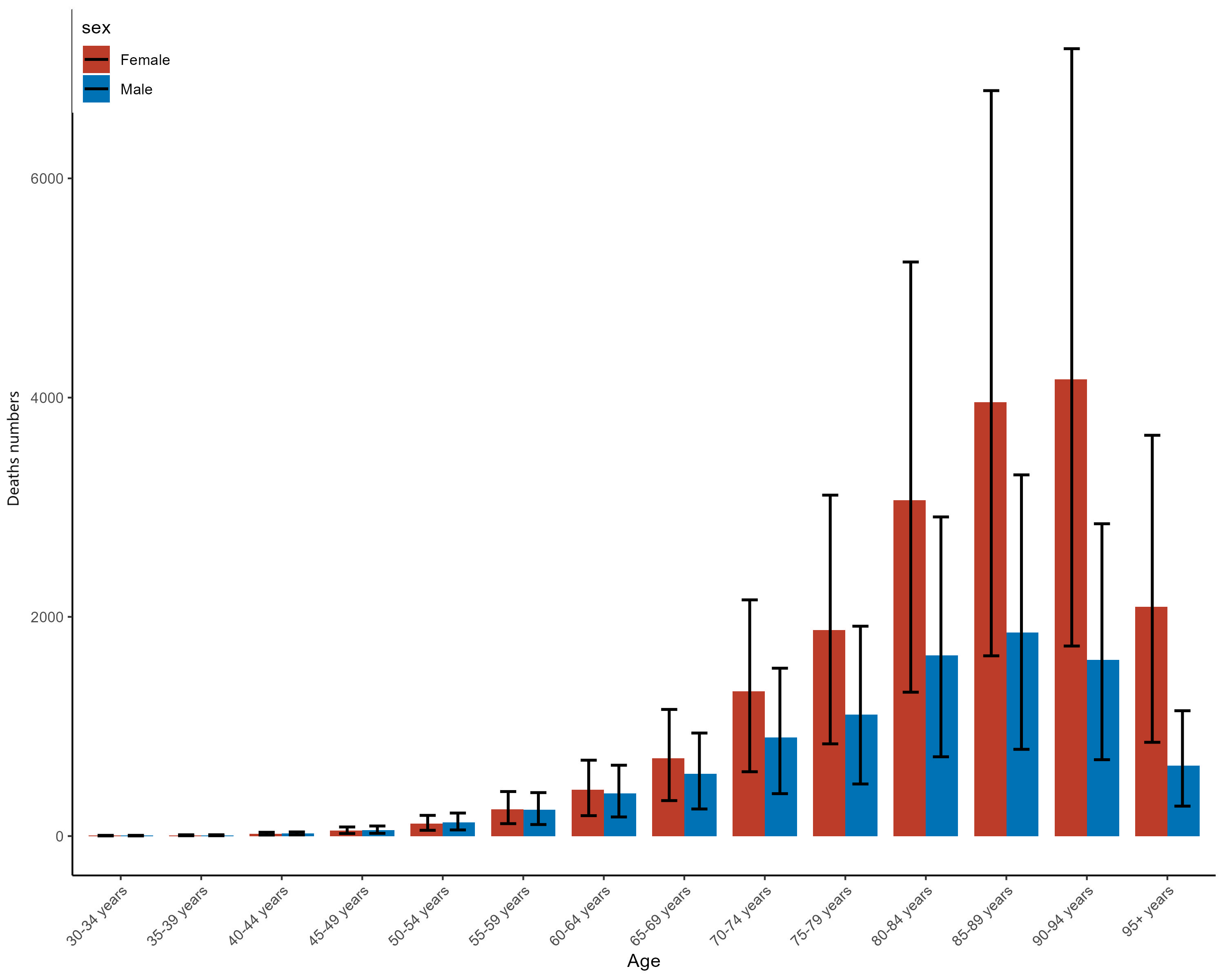

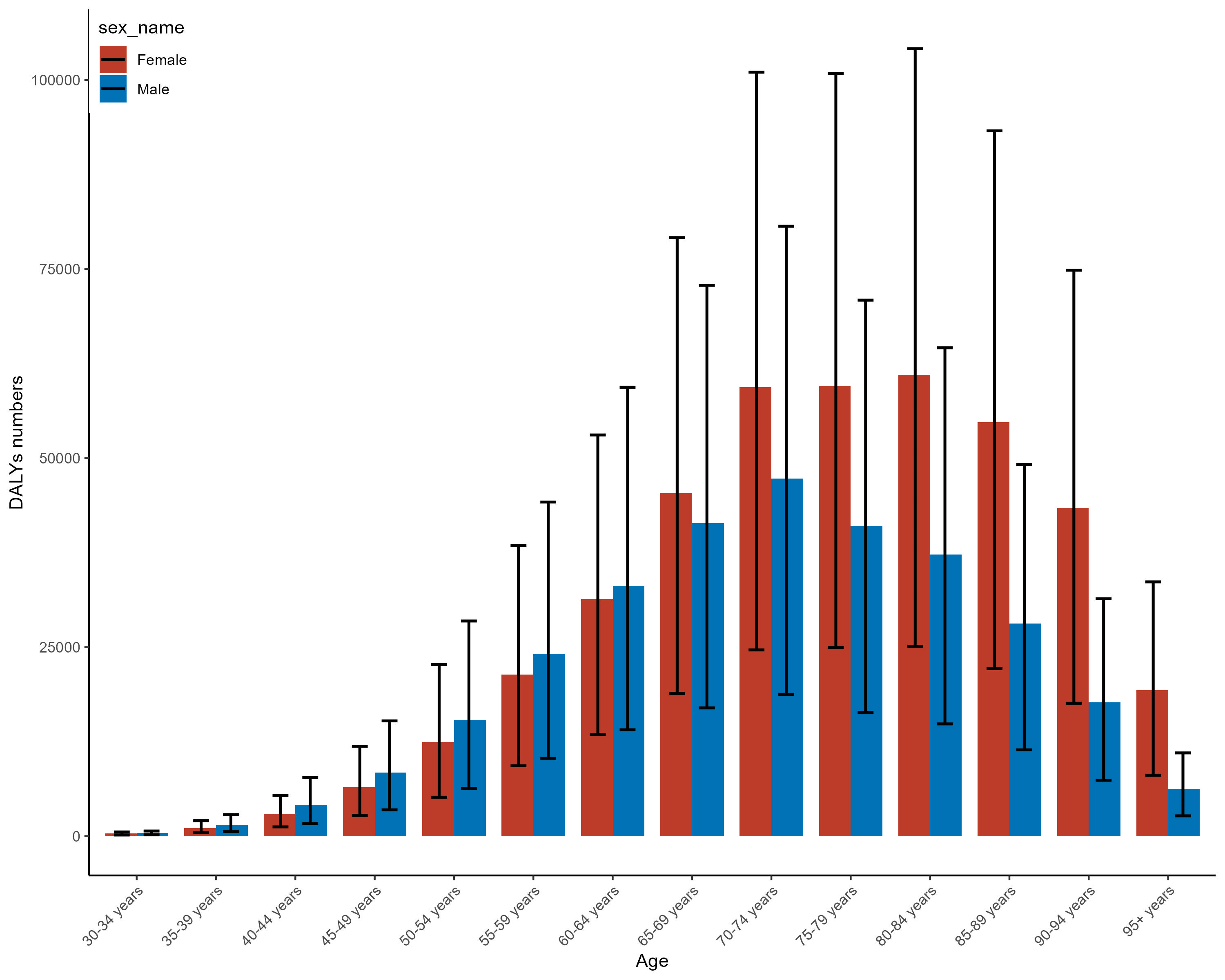


(D)


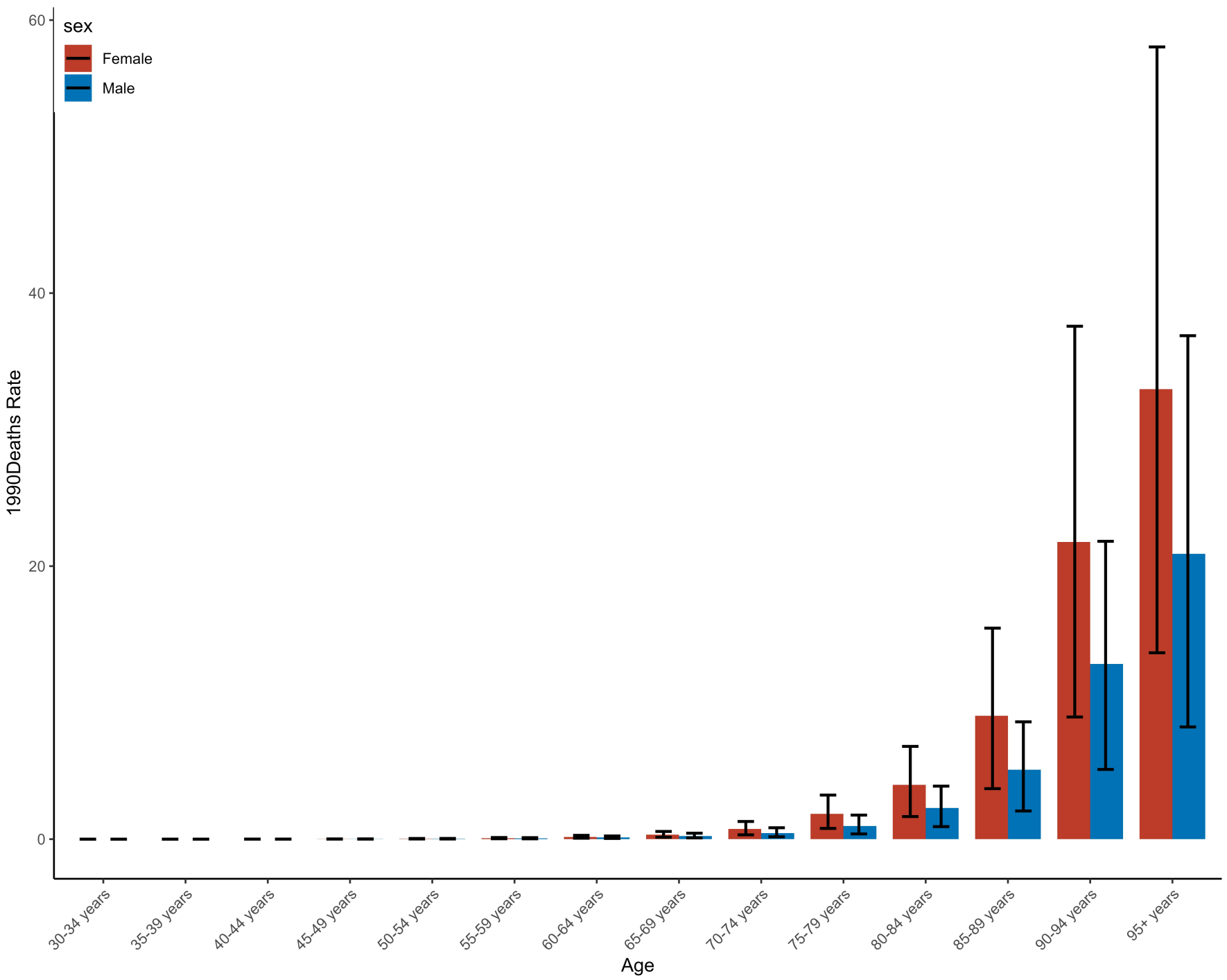

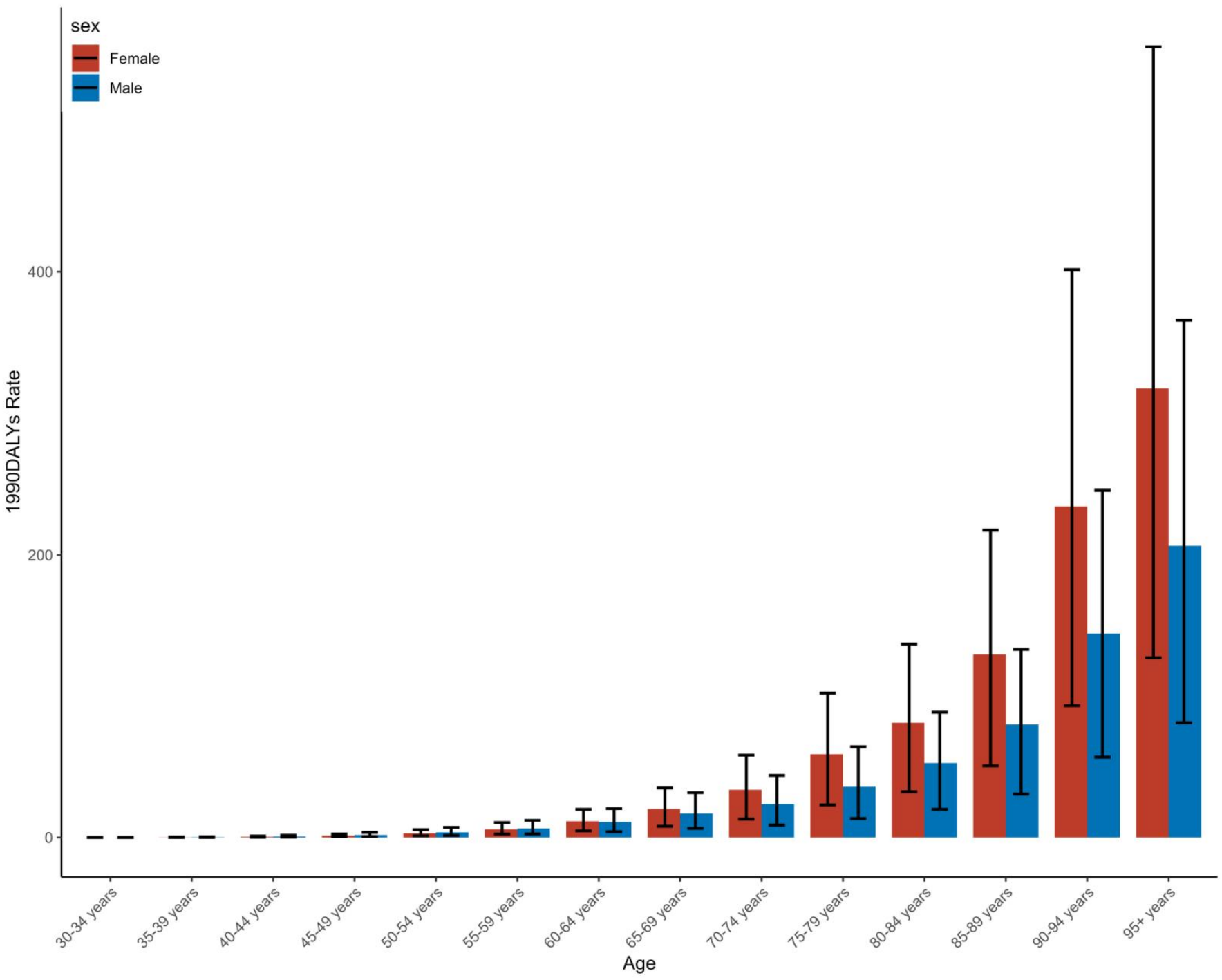


**Supplementary Figure S9.** The gender and age distribution of HBMI-related AF/AFL burden in 1990 and 2021. (A) Deaths and DALYs rates (per 100 000 persons) in 21 GBD world regions, by sex, in 1990 and 2021. (B) Global rate of deaths and DALYs in different age stratifications, by sex, in 2021. (C) Global numbers of deaths and DALYs in different age stratifications, by sex, in 2021. (D) Global rate of deaths and DALYs in different age stratifications, by sex, in 1990. Abbreviations as in Figure 1.


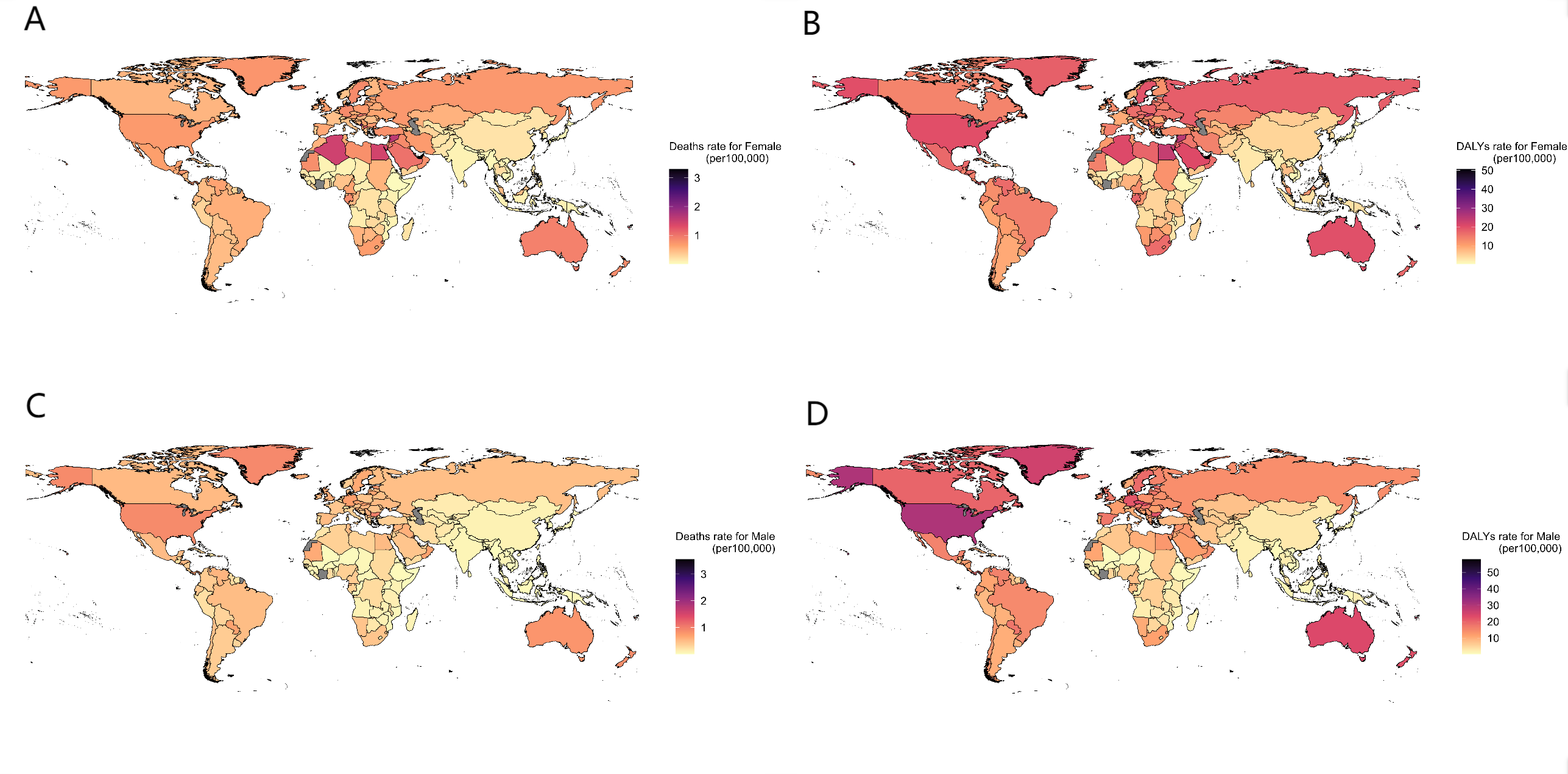


**Supplementary Figure S10.** Burden of atrial fibrillation/atrial flutter (AF/AFL) attributable to high body mass index (HBMI) across 204 countries and regions in 2021, by gender. Mortality and disability-adjusted life year (DALY) rates among female (A & C) and male (B & D) populations across 204 countries and regions in 2021. Abbreviations are the same as in Figure S1


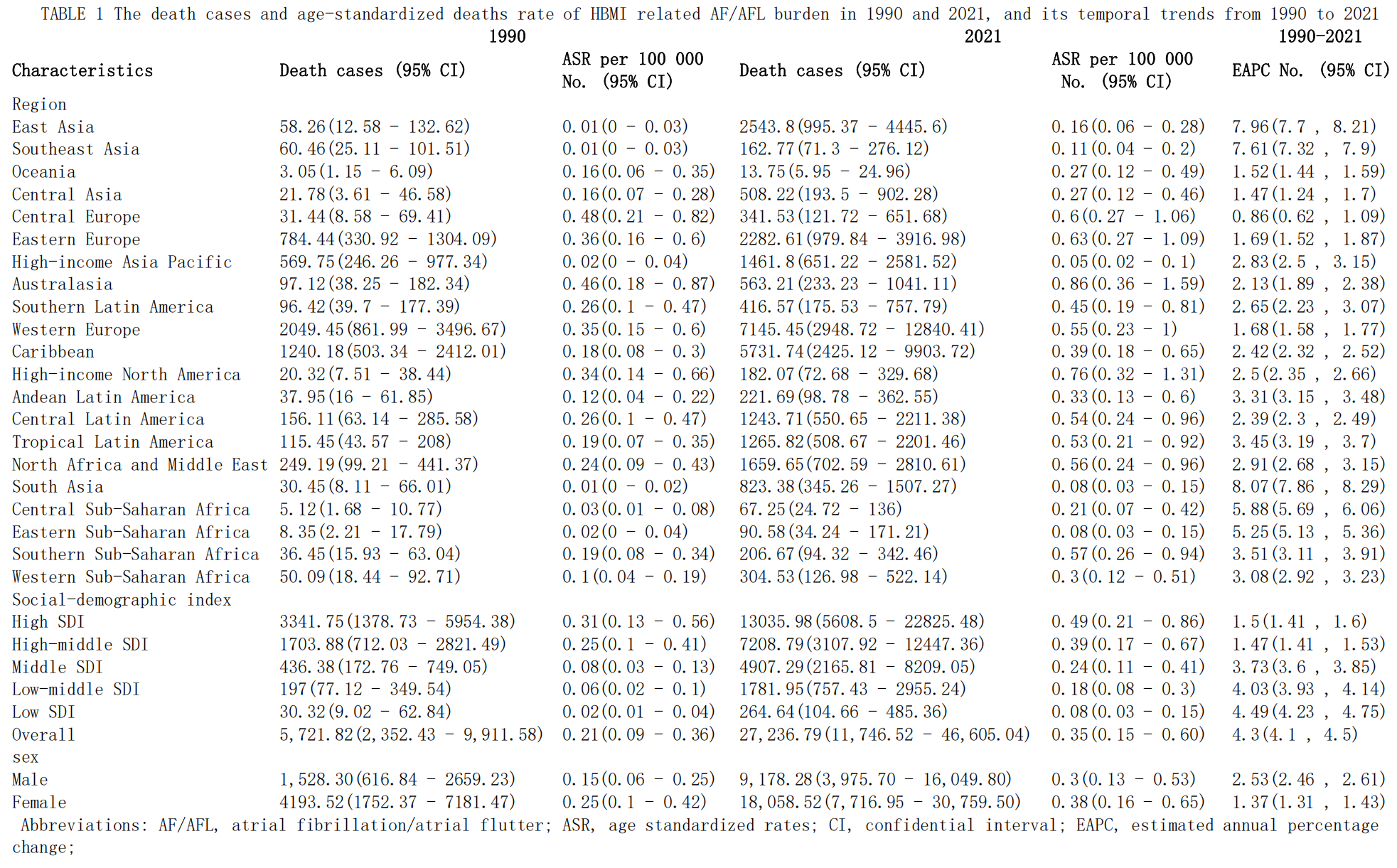


**Supplementary TABLE 1** The death cases and age-standardized deaths rate of HBMI related AF/AFL burden in 1990 and 2021, and its temporal trends from 1990 to 2021


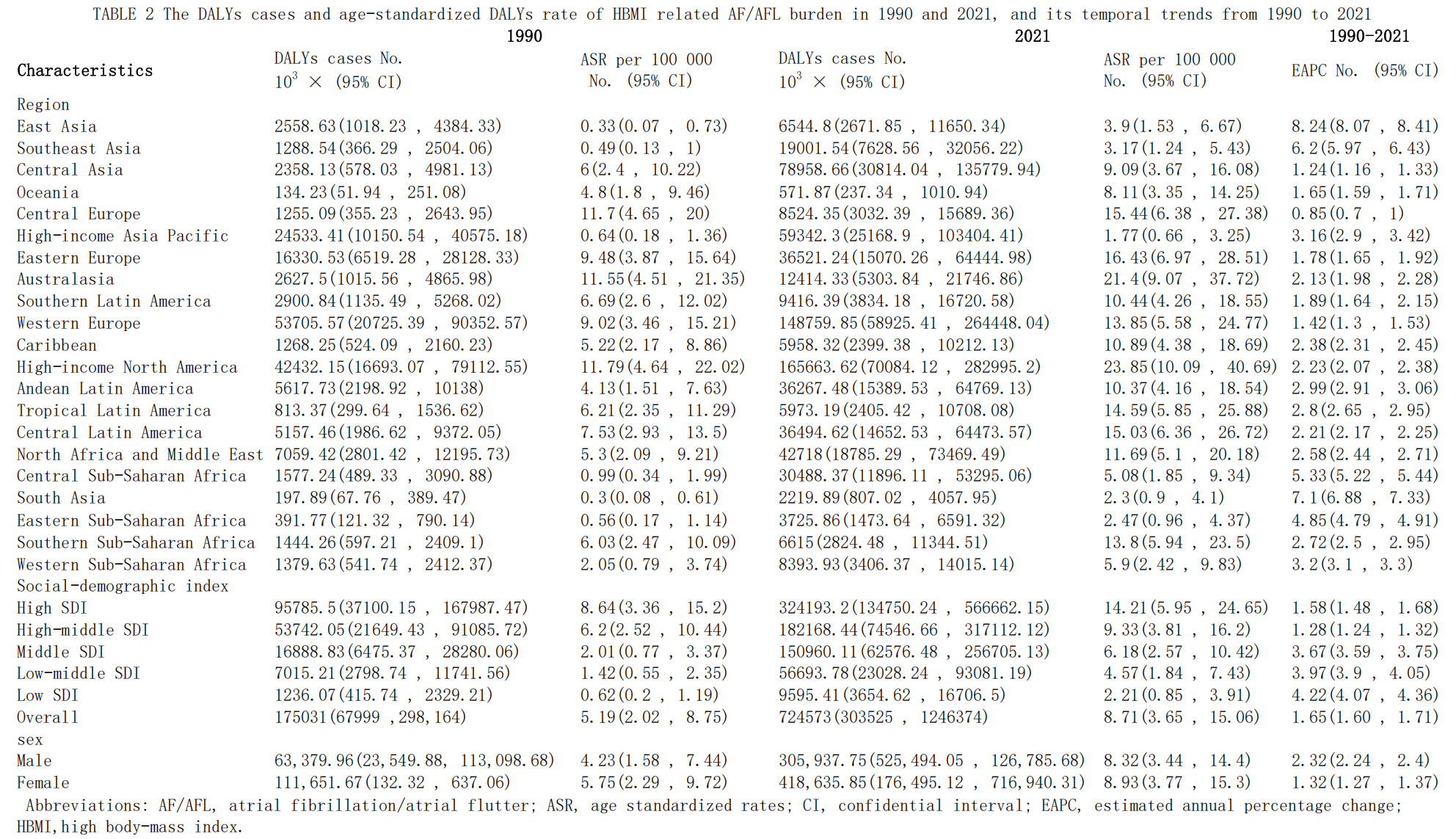


**Supplementary TABLE 2** The DALYs cases and age-standardized DALYs rate of HBMI related AF/AFL burden in 1990 and 2021, and its temporal trends from 1990 to 2021
