# Supplementary figures and images for: Global burden of atrial fibrillation/flutter attributable to a high body mass index (HBMI) from 1990–2021
Source: BMC Cardiovasc Disord. 2025 Oct 14;25:741. doi: 10.1186/s12872-025-05125-5 (PMC12522503; doi:10.1186/s12872-025-05125-5)

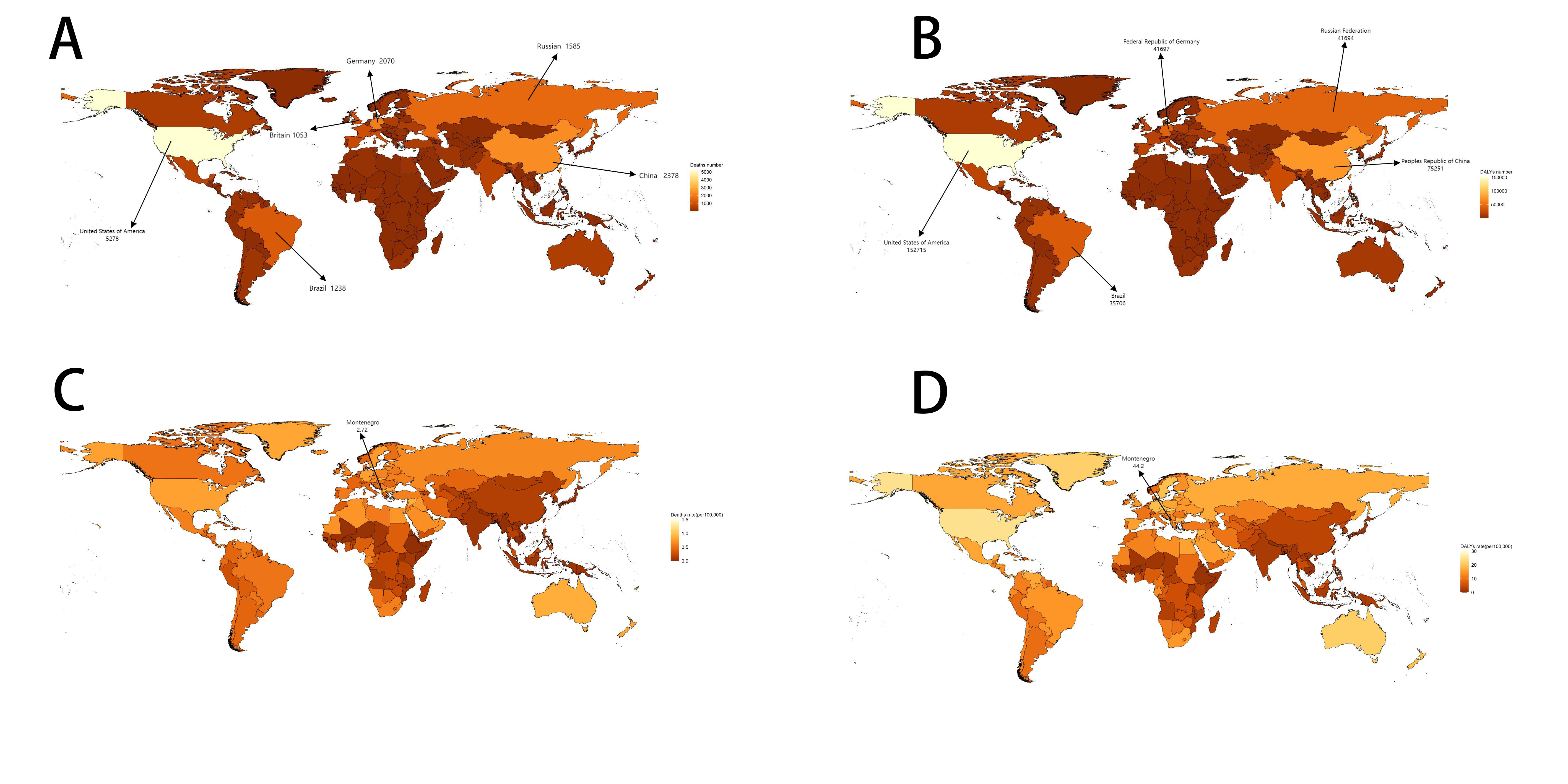

Supplement: Supplementary file 2 — Supplementary Material 2 [file 12872_2025_5125_MOESM2_ESM.zip › FIGURE 1.png]

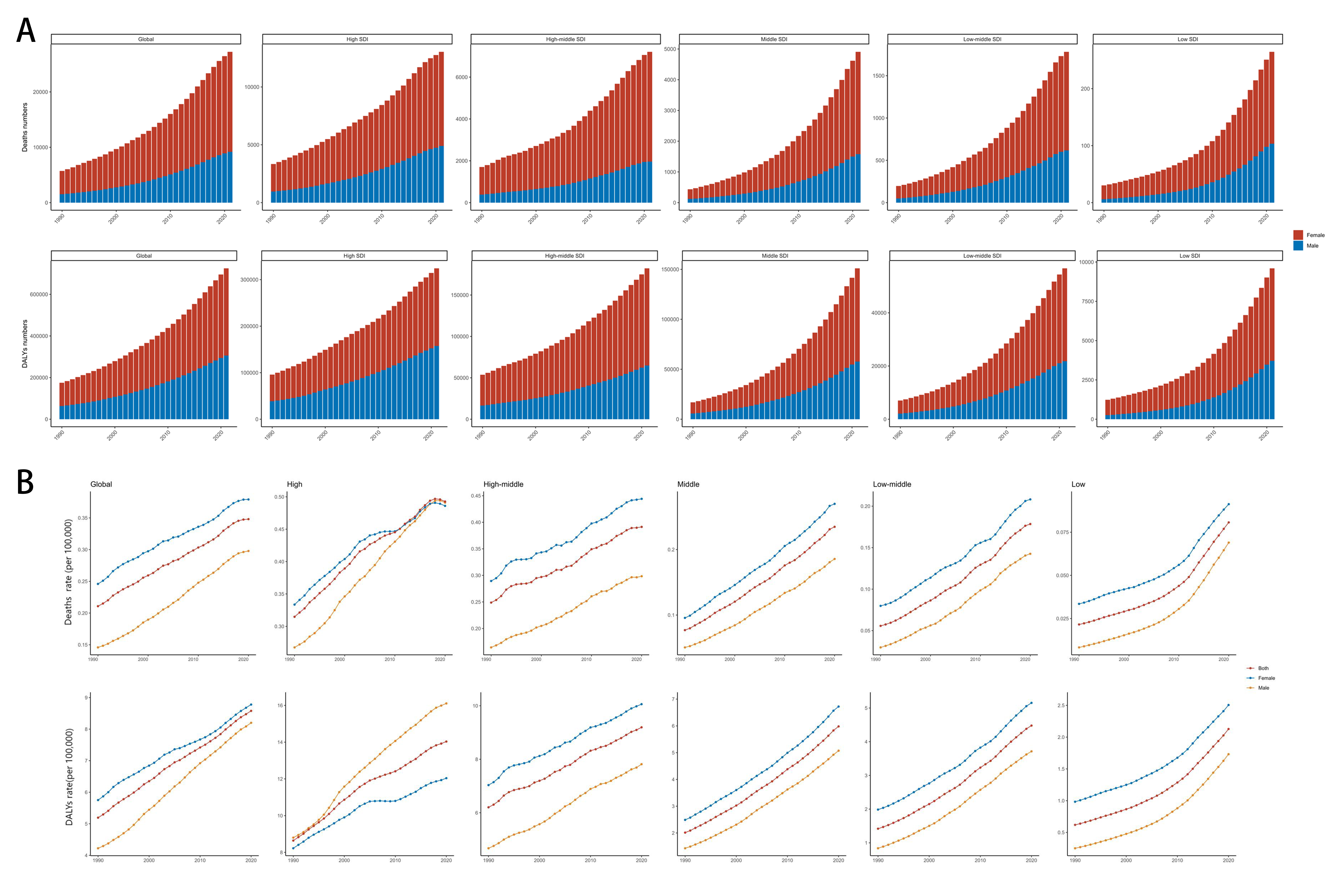

Supplement: Supplementary file 2 — Supplementary Material 2 [file 12872_2025_5125_MOESM2_ESM.zip › FIGURE 2.png]

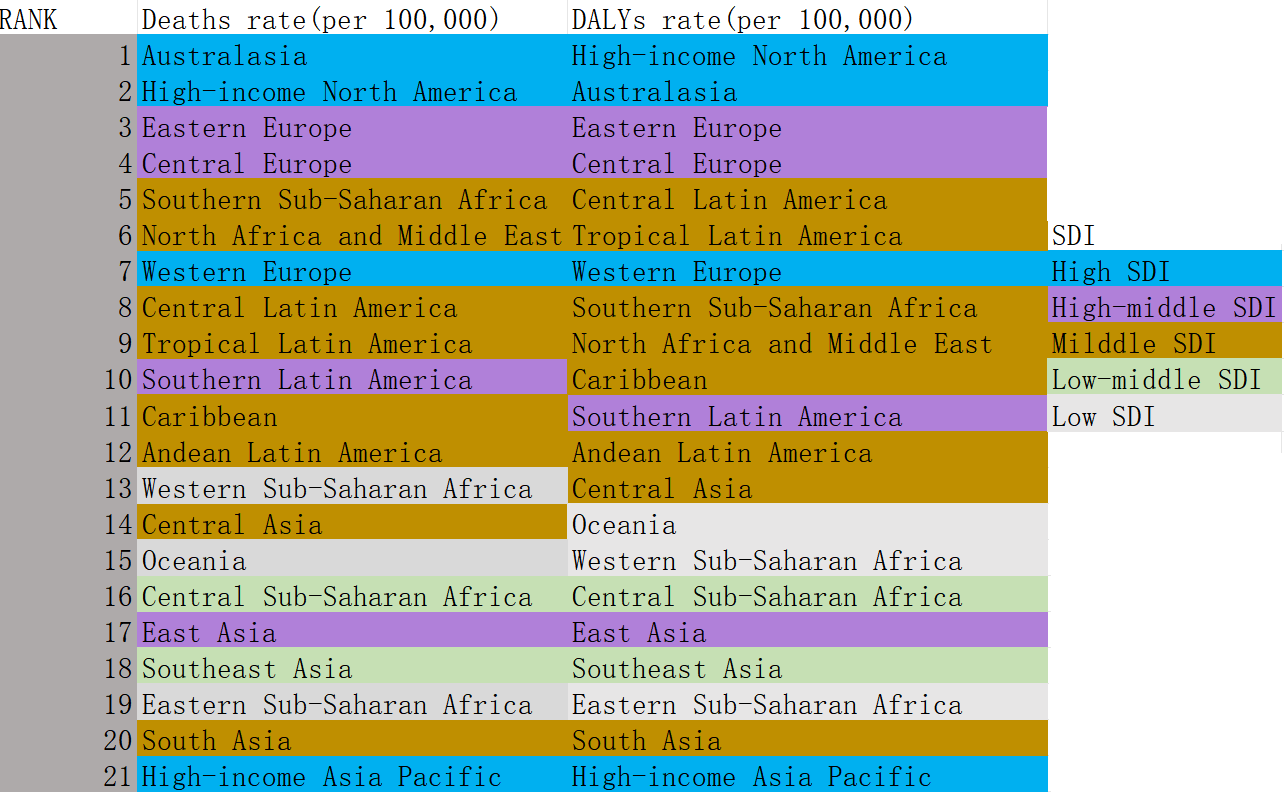

Supplement: Supplementary file 2 — Supplementary Material 2 [file 12872_2025_5125_MOESM2_ESM.zip › Supplementary Figure S1.png]

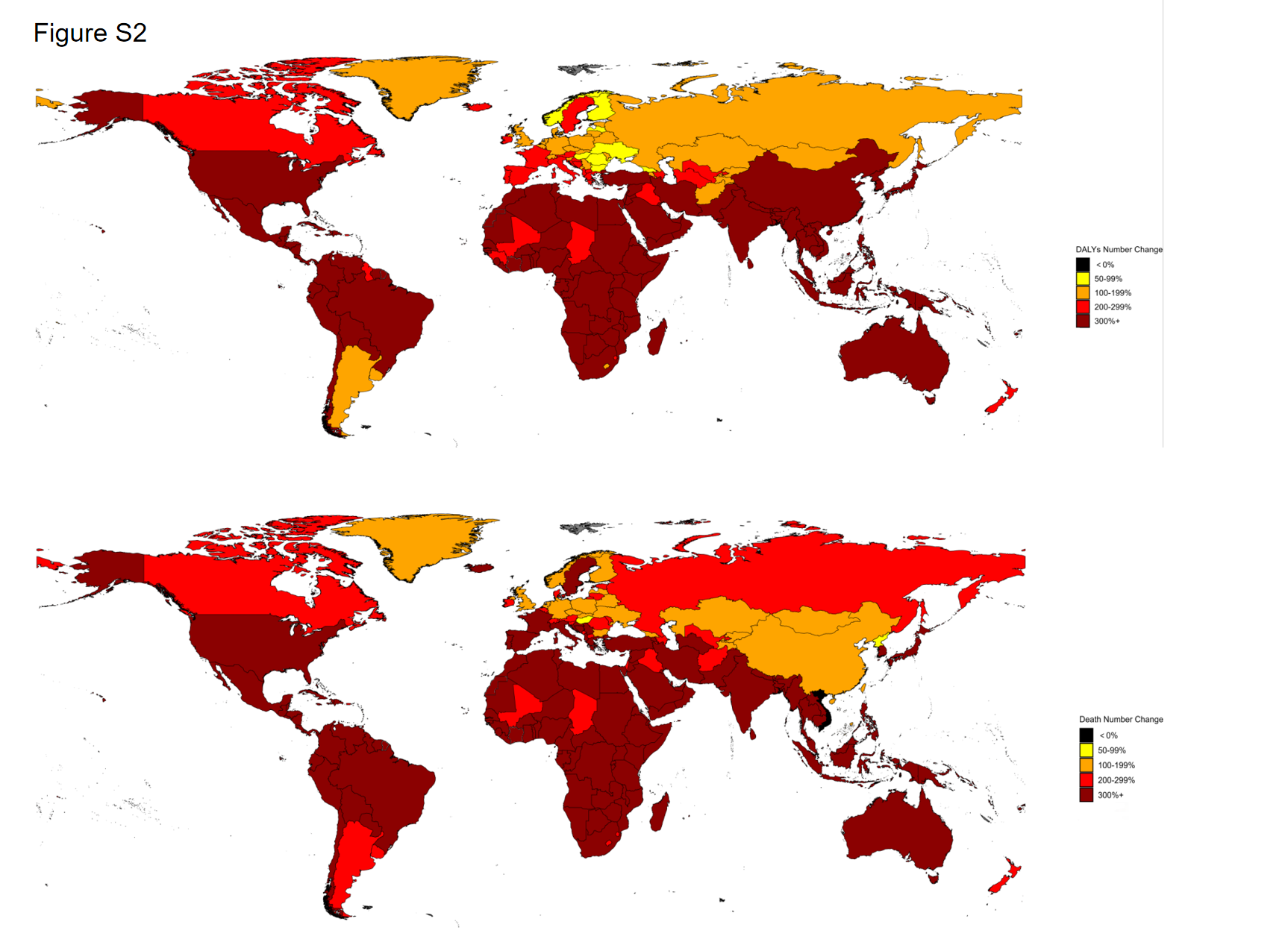

Supplement: Supplementary file 2 — Supplementary Material 2 [file 12872_2025_5125_MOESM2_ESM.zip › Supplementary Figure S2.png]

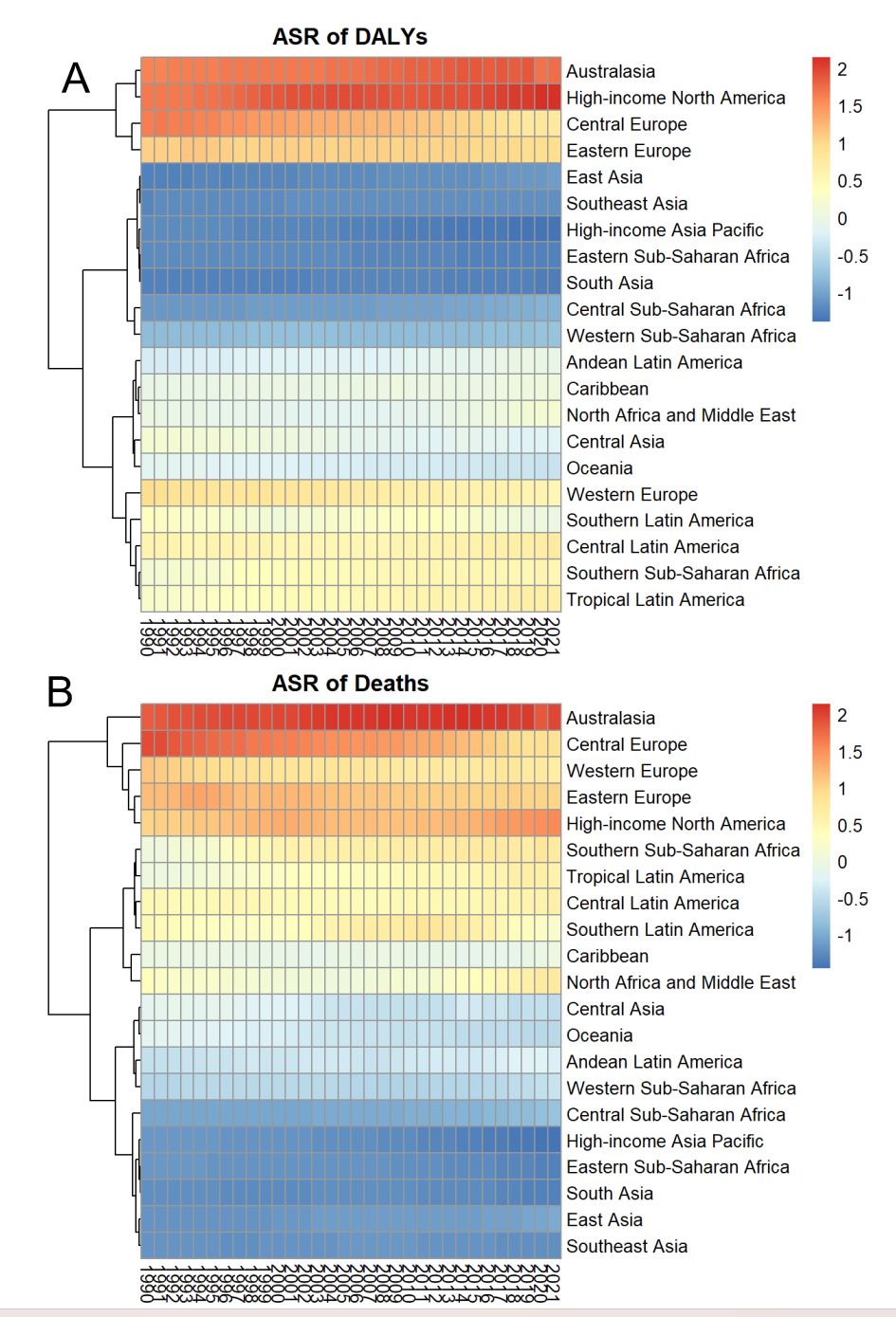

Supplement: Supplementary file 2 — Supplementary Material 2 [file 12872_2025_5125_MOESM2_ESM.zip › Supplementary Figure S3.png]

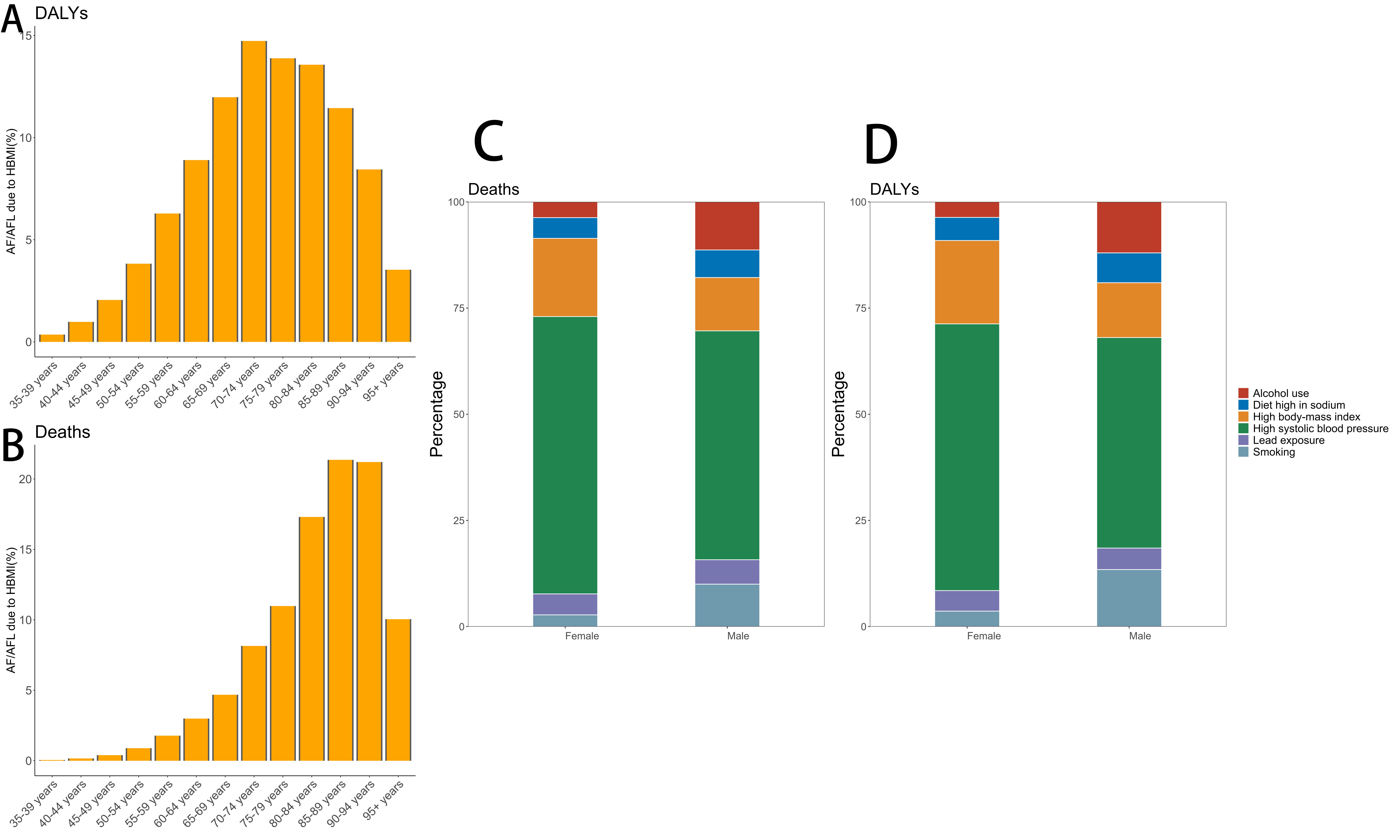

Supplement: Supplementary file 2 — Supplementary Material 2 [file 12872_2025_5125_MOESM2_ESM.zip › Supplementary Figure S7.png]

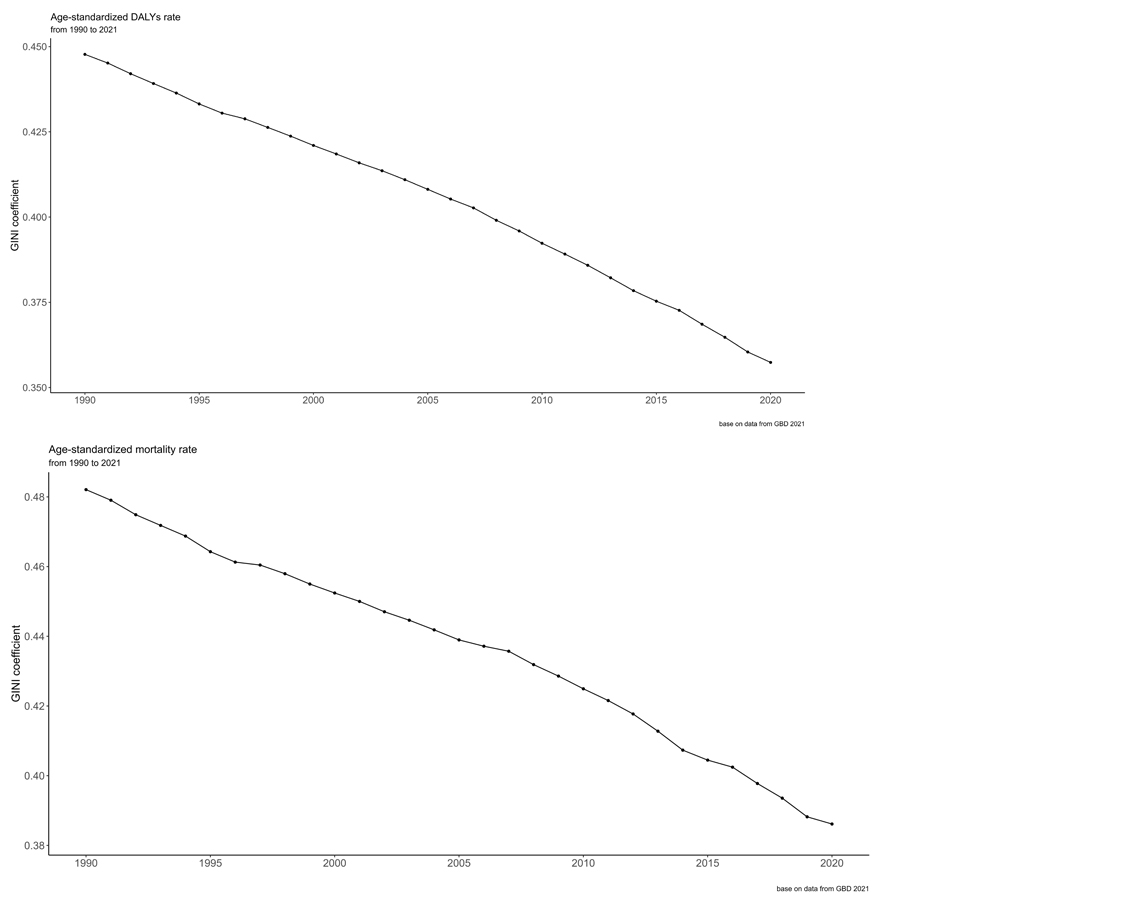

Supplement: Supplementary file 2 — Supplementary Material 2 [file 12872_2025_5125_MOESM2_ESM.zip › Supplementary Figure S8.png]

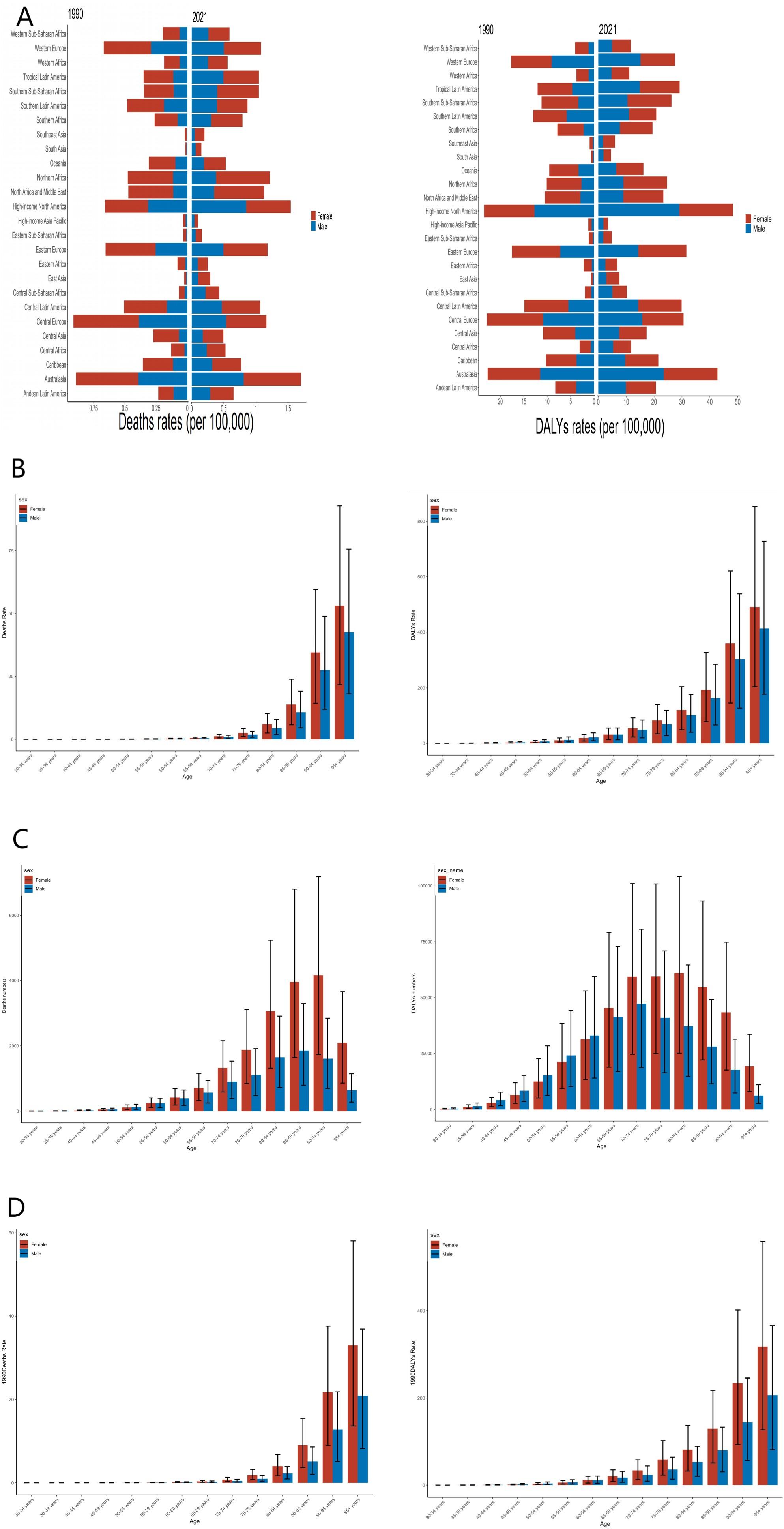

Supplement: Supplementary file 2 — Supplementary Material 2 [file 12872_2025_5125_MOESM2_ESM.zip › Supplementary Figure S9.jpg]

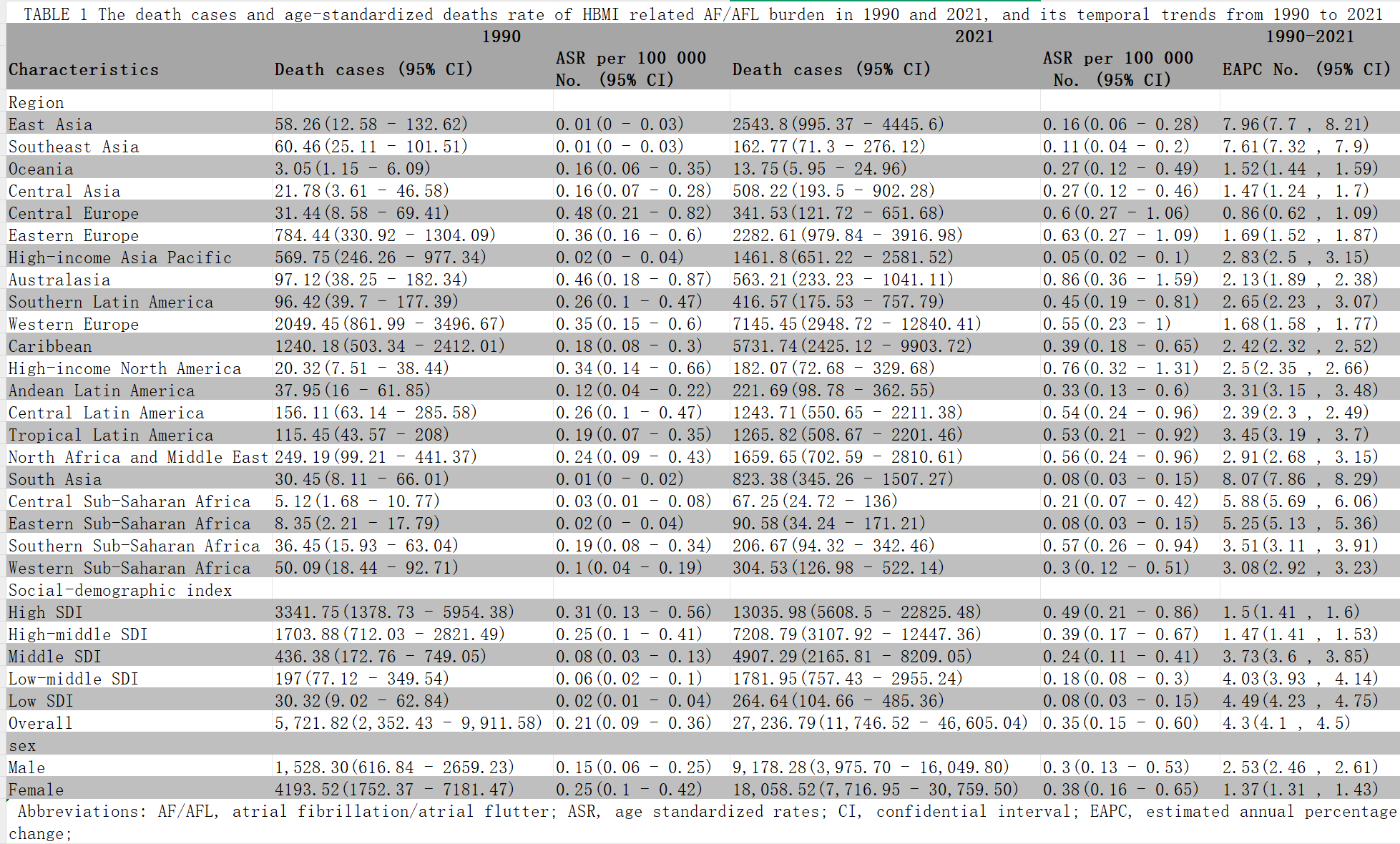

Supplement: Supplementary file 2 — Supplementary Material 2 [file 12872_2025_5125_MOESM2_ESM.zip › Supplementary TABLE 1.png]

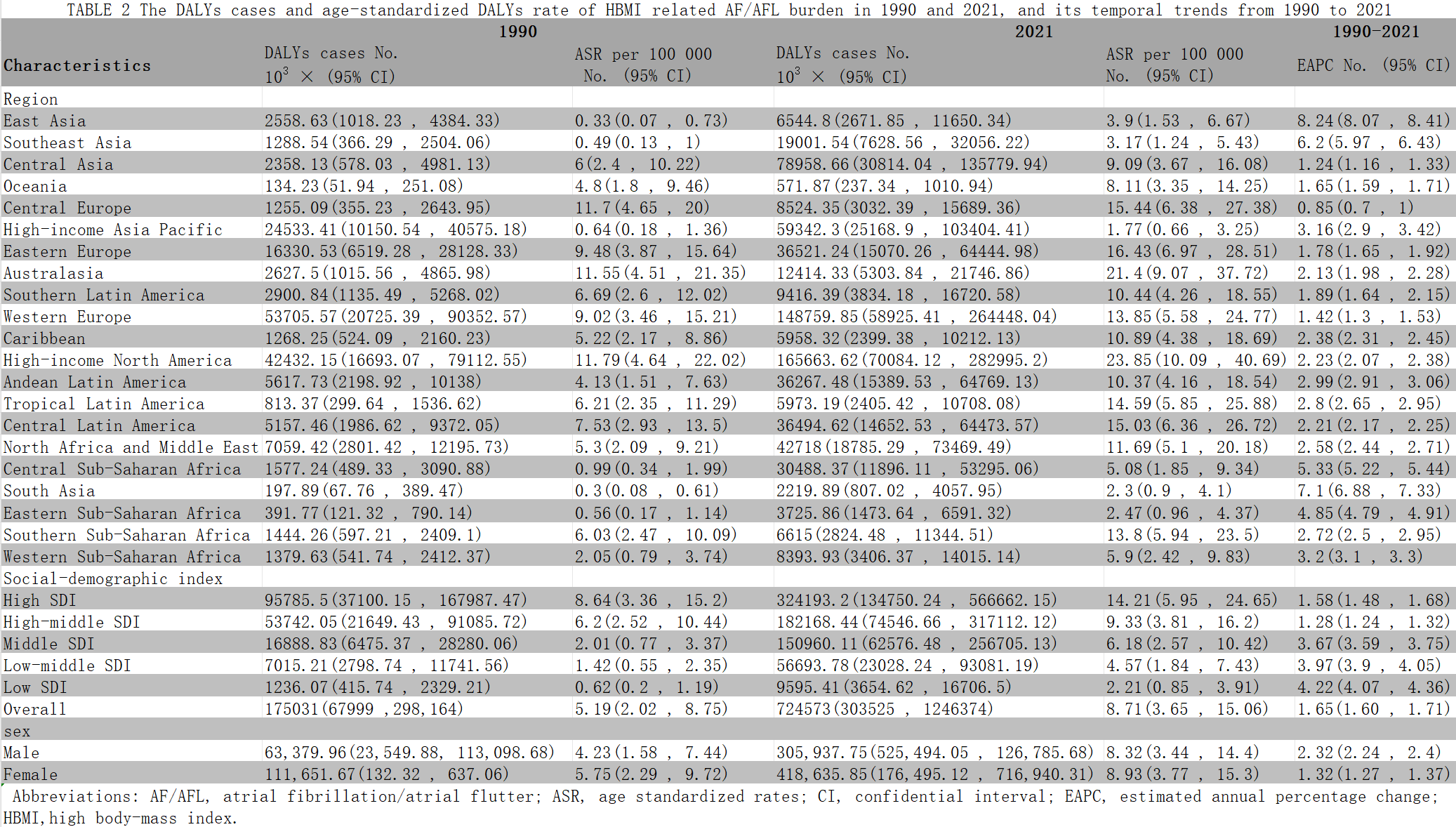

Supplement: Supplementary file 2 — Supplementary Material 2 [file 12872_2025_5125_MOESM2_ESM.zip › Supplementary TABLE 2.png]
